# Identification of immune correlates of fatal outcomes in critically ill COVID-19 patients

**DOI:** 10.1371/journal.ppat.1009804

**Published:** 2021-09-16

**Authors:** Jonathan Youngs, Nicholas M. Provine, Nicholas Lim, Hannah R. Sharpe, Ali Amini, Yi-Ling Chen, Jian Luo, Matthew D. Edmans, Panagiota Zacharopoulou, Wentao Chen, Oliver Sampson, Robert Paton, William J. Hurt, David A. Duncan, Anna L. McNaughton, Vincent N. Miao, Susannah Leaver, Duncan L. A. Wyncoll, Jonathan Ball, Philip Hopkins, Oxford Immunology Network Covid-19 response T cell Consortium, Oxford Protective T cell Immunology for COVID-19 (OPTIC) Clinical team, Donal T. Skelly, Eleanor Barnes, Susanna Dunachie, Graham Ogg, Teresa Lambe, Ian Pavord, Alex K. Shalek, Craig P. Thompson, Luzheng Xue, Derek C. Macallan, Philip Goulder, Paul Klenerman, Tihana Bicanic

**Affiliations:** 1 Institute for Infection & Immunity, St. George’s University of London, London, United Kingdom; 2 Clinical Academic Group in Infection and Immunity, St. George’s Hospital NHS Trust, London, United Kingdom; 3 Peter Medawar Building for Pathogen Research, Nuffield Department of Medicine, University of Oxford, Oxford, United Kingdom; 4 Translational Gastroenterology Unit, Nuffield Department of Medicine, University of Oxford, Oxford, United Kingdom; 5 Jenner Institute, University of Oxford, Oxford, United Kingdom; 6 MRC Human Immunology Unit, Weatherall Institute of Molecular Medicine, University of Oxford, Oxford, United Kingdom; 7 Respiratory Medicine Unit, and Oxford NIHR Biomedical Research Centre, University of Oxford, Oxford, United Kingdom; 8 Diamond Light Source, Harwell Science and Innovation Campus, Didcot, United Kingdom; 9 Institute for Medical Engineering and Science, Department of Chemistry, and Koch Institute for Integrative Cancer Research, Massachusetts Institute of Technology, Cambridge, Massachusetts, United States of America; 10 Broad Institute of MIT and Harvard, Cambridge, Massachusetts, United States of America; 11 Ragon Institute of MGH, MIT, and Harvard, Cambridge, Massachusetts, United States of America; 12 Intensive Care Medicine, St George’s University Hospital NHS Foundation Trust, London, United Kingdom; 13 Intensive Care Medicine, Guy’s and St Thomas’ Hospital NHS Foundation Trust, London, United Kingdom; 14 Centre for Human & Applied Physiological Sciences, School of Basic & Medical Biosciences, Faculty of Life Sciences, & Medicine, King’s College, London, United Kingdom; The Peter Doherty Institute and Melbourne University, AUSTRALIA

## Abstract

Prior studies have demonstrated that immunologic dysfunction underpins severe illness in COVID-19 patients, but have lacked an in-depth analysis of the immunologic drivers of death in the most critically ill patients. We performed immunophenotyping of viral antigen-specific and unconventional T cell responses, neutralizing antibodies, and serum proteins in critically ill patients with SARS-CoV-2 infection, using influenza infection, SARS-CoV-2-convalescent health care workers, and healthy adults as controls. We identify mucosal-associated invariant T (MAIT) cell activation as an independent and significant predictor of death in COVID-19 (HR = 5.92, 95% CI = 2.49–14.1). MAIT cell activation correlates with several other mortality-associated immunologic measures including broad activation of CD8^+^ T cells and non-Vδ2 γδT cells, and elevated levels of cytokines and chemokines, including GM-CSF, CXCL10, CCL2, and IL-6. MAIT cell activation is also a predictor of disease severity in influenza (ECMO/death HR = 4.43, 95% CI = 1.08–18.2). Single-cell RNA-sequencing reveals a shift from focused IFNα-driven signals in COVID-19 ICU patients who survive to broad pro-inflammatory responses in fatal COVID-19 –a feature not observed in severe influenza. We conclude that fatal COVID-19 infection is driven by uncoordinated inflammatory responses that drive a hierarchy of T cell activation, elements of which can serve as prognostic indicators and potential targets for immune intervention.

## Author summary

We examined the immune abnormalities linked to critical illness and death in COVID-19 patients on ICU, performing immunophenotyping of viral antigen-specific and unconventional T cell responses, together with studies of neutralizing antibodies, and serum proteins. We compared these findings to a parallel set of patients with severe influenza. From this screen we identified mucosal-associated invariant T (MAIT) cell activation as an independent and significant predictor of death in COVID-19. MAIT cell activation correlated with several other mortality-associated immunologic measures including elevated levels of cytokines and chemokines, such as GM-CSF and CXCL10. MAIT cell activation is also a predictor of disease severity in influenza. Single-cell RNA-sequencing revealed a shift from focused IFNα-driven signals in COVID-19 ICU patients who survive to broad pro-inflammatory responses in fatal COVID-19 –a feature not observed in severe influenza. Overall we observed key potential biomarkers and targetable pathways in critical viral illness, many shared between influenza and COVID-19 and some unique to each infection.

## Introduction

The disease course of COVID-19 is highly variable between individuals: approximately 15% of patients develop severe disease requiring intensive care unit (ICU) admission, and, of these, approximately 50% will require invasive mechanical ventilation [1]. Several studies suggest that the spectrum of clinical symptoms strongly mirrors a gradient of immune activation characterized by elevated levels of inflammatory cytokines [2–4]. Paradoxically, in severe disease some immune responses are downregulated. Type I interferon (IFN) responses, for example, are attenuated in those patients with the most severe disease and the presence of anti-IFN auto-antibodies or genetic defects in type I IFN pathways result in worse outcomes in a subset of patients [5–9]. However, one area that has remained relatively unexplored is an understanding of the immunological factors associated with death in critically ill COVID-19 patients, i.e. those requiring mechanical ventilation, in whom mortality remains as high as 30–40% [1,10]. To date, most immunology studies have been underpowered to study these responses in the context of fatal disease [2,3], limiting our understanding of how immune responses contribute or protect from fatal outcomes.

While T cell responses are essential for the control and clearance of viral infection, such as influenza virus [11], the exact role of T cell responses in SARS-CoV-2 infection remains unclear. Several studies examining COVID-19 patients have reported increased antiviral T cell responses in patients with severe (requiring ICU admission) as opposed to moderate disease (requiring hospitalization without ICU admission) [12–14]. However, other studies found impaired and hypo-functional T cell responses in the context of severe illness requiring ICU admission [15,16]. All of the aforementioned studies reported reduced T cell poly-functionality, which is postulated to be important for viral control [17].

Antibodies also have a critical role in control and protection from viral infections, such as influenza [18]. However, the role of antibodies in control of SARS-CoV-2 infection is currently ambiguous. Severity of infection is correlated with magnitude of spike- and receptor binding domain (RBD)-specific antibody titers, and neutralizing antibody titers [19–21]. However, one report suggests that individuals with severe disease have antibody responses that are proportionally less functional [19], suggestive that hypofunctional antibodies might prevent proper control of disease. Case reports of X-linked agammaglobulinemia (XLA) patients, who lack mature B cells, have reported heterogeneity in disease severity and longevity [22,23], which complicates our understanding on the necessity of antibody responses to control SARS-CoV-2 infection.

In sum, data are conflicting on the potential role of adaptive immune responses in contributing to or protecting from fatality in COVID-19. Furthermore, few comparisons have been made with other viral pneumonias in order to clarify what components of the immune response might be considered generic and which might be considered specific to COVID-19. Therefore, in this study, we sought to specifically investigate the association of T cell and antibody responses with fatal outcome in severe COVID-19 using a broad range of complementary techniques/approaches, taking patients with critical influenza infection as a similarly unwell comparator group.

## Materials and methods

### Ethics statement

For the flow cytometry and intracellular cytokine staining, subjects comprised the 41 COVID, 18 FLU, 12 HCW and 12 HC participants described in Table 1. The COVID and FLU patients were enrolled as part of an ongoing prospective observational study AspiFlu (ISRCTN51287266) which has national HRA (CPMS 43440/IRAS 271269) and REC (19/WA/0310) approval. The AspiFlu study sponsor is the St George’s Joint Research and Enterprise Services (JRES). The HCW and HC participants were enrolled at St. George’s University of London and University of Oxford. HCW and HC samples from University of Oxford were collected under the following ethics: “Characterisation of the Immune Response to SARS-CoV2 Infection and Correlates of Gastrointestinal Symptomatology and Microbiome” approved by the research ethics committee (REC) at Yorkshire & The Humber—Sheffield (GI Biobank Study 16/YH/0247). Samples from St. George’s University of London were collected as part of the “Effect of Ageing on Immunity” study, approved by NRES London, Chelsea (Ref 13/LO/1621). For the neutralizing antibody and Luminex assay, the same COVID and FLU cohorts were utilised alongside 15 healthy subjects from University of Oxford (5 male/10 female). The single-cell RNA sequencing cohort comprised 18 COVID-19 patients (12 from the AspiFlu study and 6 from [24]), 12 influenza patients (from the AspiFlu study) and 6 healthy controls from [24]. The study was conducted in compliance with all relevant ethical regulations for work with human participants, and according to the principles of the Declaration of Helsinki (2008) and the International Conference on Harmonization (ICH) Good Clinical Practice (GCP) guidelines. Written personal/professional consultee declarations were obtained for all patients at enrollment, followed by written informed consent where patients recovered capacity.

**Table 1:** Clinical summary of critically ill COVID-19 and influenza cohorts.

	COVID^a^ (n = 41)	FLU^b^ (n = 18)	*P* value
**Demographics**			
Age (years), median (IQR)	58 (48–65)	56 (46–61)	ns
Sex at birth, n (%)	M 26 (63) F 15 (37)	M 13 (72) F 5 (28)	ns
BAME^c^, n (%)	23 (56)	4 (22)	0.02
BMI^d^ median (IQR)	28 (25–30)	26 (23–32)	ns
Active/past smoker	11 (27)	8 (44)	ns
**Co-morbidities**			
Hypertension, n (%)	15 (37)	4 (22)	ns
Diabetes, n (%)	10 (24)	2 (11)	ns
Chronic lung disease, n (%)	6 (15)	6 (33)	ns
Chronic kidney disease, n (%)	3 (7.3)	1 (5.6)	ns
Severely immunocompromised^e^, n (%)	1 (2.4)	0 (0)	ns
Corticosteroids in 21 days pre ICU, n (%)	3 (7)	3 (17)	ns
Mean total steroids in 21 days pre ICU, (mg/kg of pred/equiv)	0.18	0.45	ns
**Parameters at ICU admission**			
Days post symptom onset until ICU admission, median (IQR)	8 (6–11)	4 (2–7)	<0.0001
Days post symptom onset until blood sampling, median (IQR)	14 (12–21)	9 (6–10)	0.001
Days post ICU admission until blood sampling, median (IQR)	6 (3–10)	3 (1–4)	0.002
ICU admission SOFA score	6 (5–8)	10 (9–14)	<0.0001
ICU admission Apache II score	12 (9–16)	20 (17–26)	<0.0001
ICU admission lymphocyte count (x10^9^/L)	0.7 (0.5–0.9)	0.6 (0.3–0.9)	ns
ICU admission NLR^f^, median (IQR)	10 (6–17)	14 (7–31)	ns
**ICU interventions & outcomes**			
ECMO^g^, n (%)	0 (0)	8 (44)	<0.0001
RRT^h^, n (%)	15 (37)	11 (61)	ns
Tocilizumab, n (%)	1 (2.4)	0 (0)	ns
Corticosteroids during ICU stay, n (%)	21 (51)	11 (61)	ns
Mean total steroids during ICU stay (mg/kg of pred/equiv)	6.3	2.7	ns
Days mechanically ventilated, median (IQR)	15 (10–25)	20 (9–33)	ns
Days on ICU, median (IQR)	17 (11–29)	24 (14–34)	ns
ICU mortality, n (%)	21 (51)	1 (4.5)	0.0009
	HCW^c^ (n = 12)	HC^d^ (n = 12)	
Age, median (IQR)	55 (38–59)	68 (34–76)	
Sex at birth, n (%)	M 5 (42) F 7 (58)	M 6 (50) F 6 (50)	
Days post symptom onset until blood sampling, median (IQR)	58 (42–68)	NA	

a, ICU (mechanically ventilated) COVID-19.

b, ICU (mechanically ventilated) Influenza.

c, Black, Asian and Minority Ethnic.

d, Body Mass Index (kg/m^2^).

e, Defined in accordance with EORTC/MSGERC host factors for invasive fungal disease. The patient listed was a hematopoietic stem cell transplant recipient.

f, neutrophil: lymphocyte ratio.

g, Extracorporeal membrane oxygenation.

h, Renal replacement therapy.

### Thawing cryopreserved PBMCs

Cryopreserved PBMCs were thawed rapidly and resuspended in 10 ml of R10 media (RPMI-1640 + 10% FBS + 1% Penicillin-Streptomycin) containing 2 μl Benzonase (≥25 U/μl; Sigma-Aldrich). Cells were washed a further time in R10 and plated at 1–2×10^6^ cells per well of a 96-well U-bottom plate.

### Flow cytometry

Cells were washed and resuspended in FACS buffer (PBS + 0.05% bovine serum albumin + 1mM EDTA). MR1 tetramer staining was performed for 40 min at room temperature. Cells were washed twice, and surface staining was for 30 min at 4°C in FACS buffer. Cells were washed twice and fixed in BD Cytofix/Cytoperm for 30 min at 4°C. After two further washes, cells were stored in FACS buffer at 4°C until acquired on a flow cytometer. All staining antibodies are listed in S1 Table.

### Intracellular cytokine staining

Intracellular cytokine staining was performed as previously described [25]. Cells were unstimulated or stimulated with overlapping peptide pools to SARS-CoV-2 spike protein (two pools: S1 and S2, 15 amino acids, overlapping by 10) or H3/N2 influenza virus NP+M1 proteins (single pool, 15 amino acids overlapping by 10). Anti-CD28 (clone CD28.2; ThermoFisher) and anti-CD49d (clone R1-2; ThermoFisher) were added to all wells. Cells were incubated for 2 h at 37°C, 5% CO_2_. Then Brefeldin A and Monensin (both BioLegend) were added, and cells were incubated for an additional 16 h at 37°C, 5% CO_2_. Cells were subsequently washed twice in FACS buffer, and surface staining was performed in FACS buffer for 30 min at 4°C. After two washes, cells were fixed and permeabilized with BD Cytofix/Cytoperm (BD Biosciences) for 30 min at 4°C. Cells were washed twice with 1x BD Perm/Wash buffer and intracellular staining was performed for 30 min at 4°C. After two further washes, cells were transferred to FACS buffer and stored at 4°C until sample acquisition. All staining antibodies are listed in S2 Table.

### SARS-CoV-2 pseudotyped neutralization assay

A lentivirus-based pseudotyped virus system was used to display the SARS-CoV-2 spike protein on its surface using a synthetic codon optimised SARS-CoV-2 expression construct (NCBI reference sequence: YP_009724390.1). Virus infectivity was determined by titration on HEK 293T ACE2-plasmid transfected cells as previously described [26]. Neutralizing antibody titers were determined by endpoint two-fold serial dilutions of test samples mixed with 10^5^ relative light units (RLU) of pseudotyped virus, incubated at 37°C for two hours and then mixed with 10^4^ HEK 293T ACE2-transfected cells per well. Plates were incubated for 72 hours at 37°C and then cells were lysed and assayed for luciferase expression. Neutralization titers are expressed as Log_10_(IC50) values.

### Luminex assay

The concentrations of selected proteins in the serum samples were measured with Human Magnetic Luminex Assay Kits (Bio-techne) with 3 panels containing total 51 analytes: C-C motif ligand (CCL)2/3/4/11/17/18/19/20, CD40 Ligand (CD40L), CD163, complement component 5a (C5a), C-X-C motif chemokine ligand (CXCL)1/5/10, epidermal growth factor (EGF), basic fibroblast growth factor (FGF2), granulocyte colony-stimulating factor (G-CSF), granulocyte-macrophage colony-stimulating factor (GM-CSF), granzyme B (GrB), interferon (IFN)α/β/γ, interleukin (IL)-1α/1β/2/3/5/6/8/10/12/13/15/17A/23/33, lactoferrin (LF), Lipocalin-2 (LCN2), Lymphotoxin-alpha (LT-α), macrophage colony-stimulating factor (M-CSF), Myeloperoxidase (MPO), beta-nerve growth factor (β-NGF), Oncostatin M (OSM), S100 calcium-binding protein A9 (S100A9), stem cell growth factor (SCGF), tissue factor (TF), tissue factor pathway inhibitor (TFPI), transforming growth factor alpha (TGF-α), Thrombopoietin (THPO), tumor necrosis factor (TNF) and triggering receptor expressed on myeloid cells 1 (TREM-1). The assays were conducted according to the manufacturer’s instruction. Results were obtained with a Bio-Rad Bio-Plex 200 System.

### Flow cytometry data analysis and statistics

Flow cytometry data was acquired on either a BD LSRII flow cytometer (BD Biosciences) or a custom 4-laser Aurora Spectral Analyser (Cytek). Cytometry data were processed in FlowJo v. 10.6.2 (FlowJo, LLC). Flow cytometry and Luminex data were analysed in Prism v. 8.4.3 (GraphPad Software, Inc.). Statistical tests used are indicated in the appropriate figure legend. For analysis associated with mortality in COVID-19 patients, adjustment for multiple comparisons was performed using the Benjamini-Hochberg FDR calculation on a dataset containing all tested *P* values (99 parameters). The same approach was taken for the comparison of died or ECMO treatment versus not in the influenza patients (47 parameters). Because of the smaller influenza patient cohort size, no FDR value was <0.1. When *P*<0.05, the exact *P* values are reported in the figure; otherwise ns (not significant) is reported.

### Single-cell RNA-sequencing

#### Sample preparation

scRNA-seq was performed via Seq-Well S^3^ as described in [27]. 10 μl of PBMCs were mixed at a 1:1 dilution with Trypan blue and counted using a hemocytometer. Cells were then diluted in RPMI + 10% FBS to a final concentration of 75,000 cells/ml of which 200μl was used to add on top of each Seq-Well array (15,000 cells) that have been pre-loaded with mRNA capture beads (ChemGenes). The arrays were then sealed using a functionalized polycarbonate membrane and placed in an incubator at 37°C for 40 minutes. The arrays were then submerged in a lysis buffer for 20 minutes and transferred into hybridization buffer for another 40 minutes. mRNA hybridized beads were recovered from each array and resuspended in a reverse transcription mix using Maxima H Minus Reverse Transcriptase, PEG, RNase inhibitor, dNTPs and a template-switching oligonucleotide. Exonuclease digestion was then carried out, followed by second strand synthesis to recover transcripts that have failed the initial template switch. This was carried out using Klenow Fragment and dN-SMART oligonucleotides. Whole transcriptome amplification (WTA) was then performed using KAPA HiFi PCR Mastermix followed by AMPure XP SPRI bead cleanup. The WTA product was then analyzed using the Agilent D5000 Screen Tape system and quantified using Qubit High-Sensitivity DNA kit. Dilutions were carried out based on these results for library preparation via Nextera XT DNA library preparation kit. Tagmented libraries were then purified again using AMPure XP SPRI beads before pooling the libraries for each array back together. The libraries were then quantified again using Qubit High-Sensitivity DNA kit and analyzed using the Agilent D5000 ScreenTape System (expected library size ranging from 300–800 bp). Libraries from 2 arrays were then pooled and sequence together on the NextSeq using a NextSeq500/550 v2 kit (75 cycles). A paired end read structure was using with a custom read 1 primer 20 bases long (12-bp cell barcode, 8bp UMI), an 8-bp read 1 index and 50 bases of read 2 for the transcript. Data uploaded to GEO accession number GSE178404.

#### Data processing

The sequences were subsequently aligned, demultiplexed and UMI collapsed as described in [28] using hg19 as the reference genome. The cell by gene matrix was loaded into Seurat and cells with >25% reads from mitochondrial genes or fewer than 600 UMIs were excluded from further analysis. We then identified highly variable genes as a reference for integrating the data. To account for potential technical effects upon loading the publicly available dataset from Wilk et. al [24] (GEO accession number GSE150728), we applied Seurat’s integration method between “SGUL” and “Stanford” representing the two locations in which the clinical data was obtained. 2000 anchor features were identified when filtered using 200 genes as neighbors and selected using canonical correlation analysis. These anchors were used to integrate both datasets via mutual nearest neighbors. We then applied principal component analysis to generate 50 principal components. An elbow plot was subsequently constructed to identify significant PCs to be used for further dimensionality reduction and clustering. The integrated dataset was then visualized using Uniform Manifold Approximation and Projection (UMAP) using the top 20 PCs.

To identify distinct cell types, we used the Wilcoxon rank-sum test (FindAllMarkers) available in Seurat and identified the top 30 genes within each cluster, or used DirichletReg [29]. Each cluster was annotated based on cluster-specific genes identified in the literature [30–32]. Module scores were assessed using a curated list of genes associated with type-I IFN response and scored based on the average expression of each gene of interest subtracted by the expression of similar control genes that have been binned together (AddModuleScore). Average expression was calculated based on the normalized counts for the gene of interest (AverageExpression).

#### Differential expression analysis

To identify differentially expressed genes while simultaneously accounting for confounders that may influence results, we subset the data on major cell types and ran SCTransform on the raw counts to remove variation associated with cellular complexity (number of UMIs). We then conducted differential expression analysis using MAST [33] and the following model:

Design∼percent.mito+Sex+Age+DaysPostSymptomsOnset

*P* values were adjusted for multiple comparisons using Bonferroni correction. Differentially expressed genes were filtered at 0.25 log-fold change and p-adjusted value of 0.05.

To further validate the robustness of these findings, we repeated the differential expression tests repeated the analysis within a subset of each categorical variable within each group to ensure that the findings from the broader dataset could be recapitulated. Specifically, we subset our data based on Age, Sex and Days Post Symptoms Onset. Lastly, to exclude the potential of UMI count as an influence, we downsampled the dataset to match for equivalent distribution of complexity metrics before running differential expression under the same parameters.

Module scores were assessed using a curated list of genes associated with type-I IFN response and scored based on the average expression of each gene of interest subtracted by the expression of similar control genes that have been binned together (AddModuleScore).

#### Geneset Enrichment Analysis (GSEA)

Gene Set Enrichment Analysis (GSEA) [34] was performed using unfiltered differentially expressed genes that were pre-ranked by average log fold-change and analysed via GSEA version 4.0.3 using the REACTOME database. Significant genesets were filtered based on p-adjusted value less than 0.05.

## Results

### Description of the patient cohort

**Fig 1:**
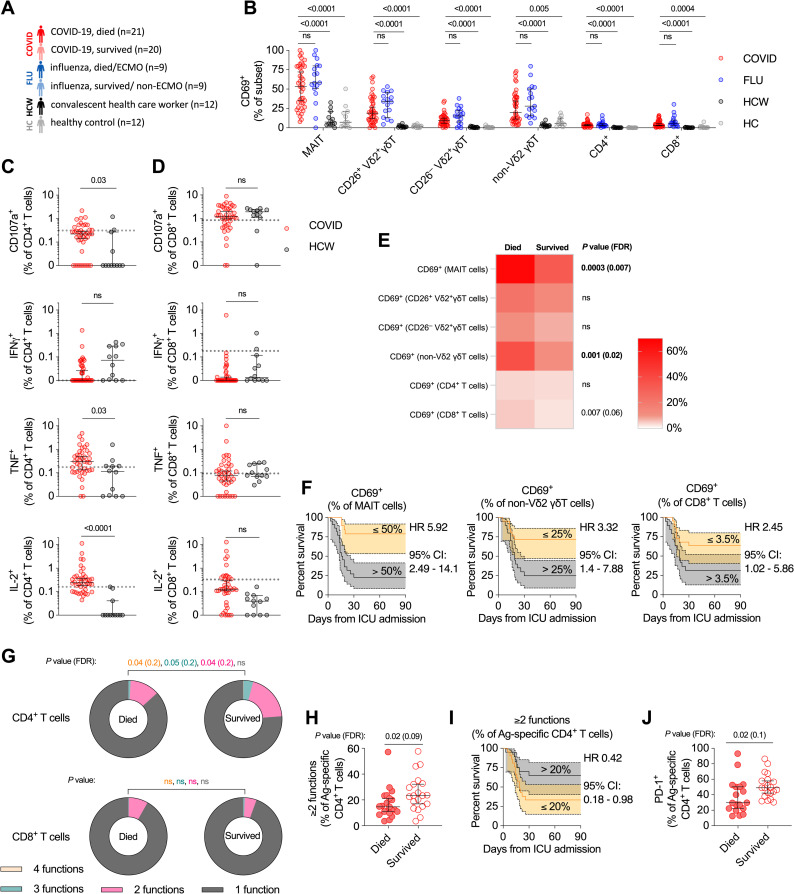
Relationship between T cell activation and mortality in critically ill COVID-19 patients. **(A)** Schematic of the study cohort. All COVID-19 and influenza patients were critically ill. Convalescent health care workers had mild disease. Samples from healthy controls were collected pre-pandemic. **(B)** Direct ex vivo measurement of general activation of each T cell subset. **(C-D)** Quantification of spike-specific CD4^+^ T cells (C) and CD8^+^ T cells (D) in acute critically ill COVID-19 patients and health care workers. Dashed line indicates the upper 95% confidence interval for responses detected in pre-pandemic healthy controls. **(E)** Median ex vivo activation level in each T cell subset of critically ill COVID-19 patients who died or survived. **(F)** Kaplan-Meier survival curves of critically ill COVID-19 patients based on relative CD69 expression on each T cell subset. **(G)** Polyfunctionality (CD107a, IFNγ, TNF, and/or IL-2) of spike-specific CD4^+^ T cells (top) and CD8^+^ T cells (bottom) between critically ill COVID-19 patients who died or survived. **(H)** Fraction of spike-specific CD4^+^ T cells that are polyfunctional (≥2 cytokines produced) in critically ill COVID-19 patients that died versus survived. **(I)** Kaplan-Meier survival curve of critically ill COVID-19 patients based on fraction of polyfunctional spike-specific CD4^+^ T cells. **(J)** PD-1 expression on spike-specific CD4^+^ T cells from critically ill COVID-19 patients who died or survived. Dots represent individual patients. Median ± 95% CI are shown. (B) Kruskal-Wallis tests with Dunn’s multiple comparison test. (A to E, G, H, and J) Mann-Whitney U-test. Benjamini-Hochberg FDR calculation was used for all statistical analyses involving associations with mortality.

To investigate the role of T cells in differential survival of critically ill COVID-19 patients, we performed detailed immune phenotyping in a cohort of 41 clinically well-characterized, mechanically ventilated, ICU COVID-19 patients (hereafter COVID) (Fig 1A). Twenty-one (51%) of these patients died during their time in ICU (Table 1). As a disease comparator, we included a cohort of mechanically ventilated, ICU patients with influenza (hereafter FLU; *n* = 18), eight of whom required additional organ support with extra-corporeal membrane oxygenation (ECMO). To contextualize responses seen in the COVID cohort, we also examined cohorts of patients convalescing from mild COVID-19 (health care workers; HCW; *n* = 12), and age-matched pre-pandemic healthy controls (HC; *n* = 12) as controls (Fig 1A and Table 1).

### Frequency and activation of T cells in critically ill COVID-19 and influenza patients

Consistent with other reports [35], lymphopenia was observed in the COVID patients (Table 1), but this did not translate into differential loss of specific CD4^+^ or CD8^+^ T cell subsets (S1A and S1B Fig). There was a loss of circulating mucosal-associated invariant T (MAIT) cells and CD26^+^ Vδ2^+^ γδT cells (S1C Fig), which are functionally similar to MAIT cells [36]. However, this was not specific to COVID-19, as loss of these cells was also observed in the FLU patients. We observed no significant loss of invariant natural killer T (iNKT) cells, consistent with one report [37] but not another [38]. No significant alteration in frequency of CD26^−^ Vδ2^+^ γδT cells or non-Vδ2 γδT cells, which are functionally distinct [39,40], was observed (S1C Fig).

We first examined general markers of T cell activation. All conventional (CD4^+^ and CD8^+^) and unconventional (MAIT, CD26^+^ Vδ2^+^ γδT, CD26^−^ Vδ2^+^ γδT, and non-Vδ2 γδT) T cell populations showed general activation as measured by CD69 upregulation (Fig 1B). MAIT cells had the highest levels of CD69 expression (median 53.3% positive versus 6% for CD8^+^ T cells). Again, this activation was not COVID-19-specific, and equivalent activation was observed in the FLU patients. Activation of iNKT cells could not be reliably assessed due to low cell numbers. CD69 expression was highly correlated between the different cell populations (S1D Fig). There have been mixed reports as to the utility of Ki-67^+^ or HLA-DR^+^CD38^+^ as markers of SARS-CoV-2-specific T cells [15,40]; we observed no significant increase in frequency of either sets of markers on T cells in our COVID cohort (S1E and S1F Fig).

### Characterization of antigen-specific T cell responses in critically ill COVID-19 patients

Direct measurement of SARS-CoV-2-specific T cells using overlapping spike peptide pools identified elevated Ag-specific CD4^+^ T cell responses in the COVID patients compared to convalescent HCWs (Fig 1C). IFNγ production was detected in only a minority of COVID patients, but strong signals for CD107a expression (as a measure of degranulation), and TNF and IL-2 production were observed (Fig 1C). Pre-existing, cross-reactive coronavirus-specific responses have been identified in a subset of SARS-CoV-2 unexposed individuals [41–43]. CD4^+^ T cells from COVID patients produced substantially more TNF and IL-2 than responses seen in pre-pandemic controls (TNF 95% CI: COVID: 0.13–0.5%, HC: 0.01–0.18%; IL-2 95% CI: COVID: 0.19–0.36%, HC: 0.06–0.16%; Fig 1C, horizontal dashed line). By contrast, spike-specific CD8^+^ T cell responses were quite weak in blood, and only CD107a expression could be unequivocally detected above pre-pandemic responses in the majority of patients (Fig 1D, horizontal dashed line). No clear pattern for IL-17A or IL-4 spike-specific responses was observed (S1G and S1H Fig). CD4^+^ T cell responses in the COVID cohort were significantly more poly-functional than CD4^+^ T cells from convalescent HCWs (median 19.3% versus 7.94% ≥2 cytokines; S1I Fig). No difference in poly-functionality of spike-specific CD8^+^ T cells was observed between COVID and HCW cohorts.

### Association of T cell responses with mortality in critically ill COVID-19 patients

We next sought to determine how these measures of T cell activation and function related to mortality in the COVID cohort. Strikingly, CD69 expression on MAIT cells, non-Vδ2 γδT cells, and conventional CD8^+^ T cells was significantly higher in patients who died versus those who survived (Fig 1E). Above median expression of CD69 on MAIT cells had the strongest association with risk of mortality in the cohort (HR = 5.92; Fig 1F). This was followed by CD69 expression on non-Vδ2 γδT cells (HR = 3.32) and conventional CD8^+^ T cells (HR = 2.45; Fig 1F).

Analysis of SARS-CoV-2 specific conventional CD4^+^ T cell and CD8^+^ T cell responses by peptide production or surrogate markers of activation, found only increased production of TNF by spike-specific CD8^+^ T cells to be associated with increased mortality (S2A, S2B, and S2C Fig).

Despite no single measure of CD4^+^ T cell function being associated with differential survival, COVID patients who survived had more poly-functional CD4^+^ T cell responses, an effect not seen with CD8^+^ T cells. (Figs 1G, 1H, 1I, and S2D). Deeper analysis of the poly-functional CD4^+^ T cell response did not reveal an obvious signature or combination of effector functions associated with survival, instead it appeared to reflect a broad overall increase in functionality (S2E and S2F Fig). Analysis of spike-specific CD4^+^ T cells revealed decreased PD-1 expression and a trend towards a reduced effector phenotype (CD45RA^−^CCR7^−^) in patients who died (Figs 1J and S2G), suggesting a less activated phenotype. Collectively, these data demonstrate impairment of Ag-specific CD4^+^ T cells in fatal COVID-19, and suggest that CD4^+^ T cells may have a protective role in this setting.

### Examination of B cell and antibody responses in critically ill COVID-19 patients

**Fig 2:**
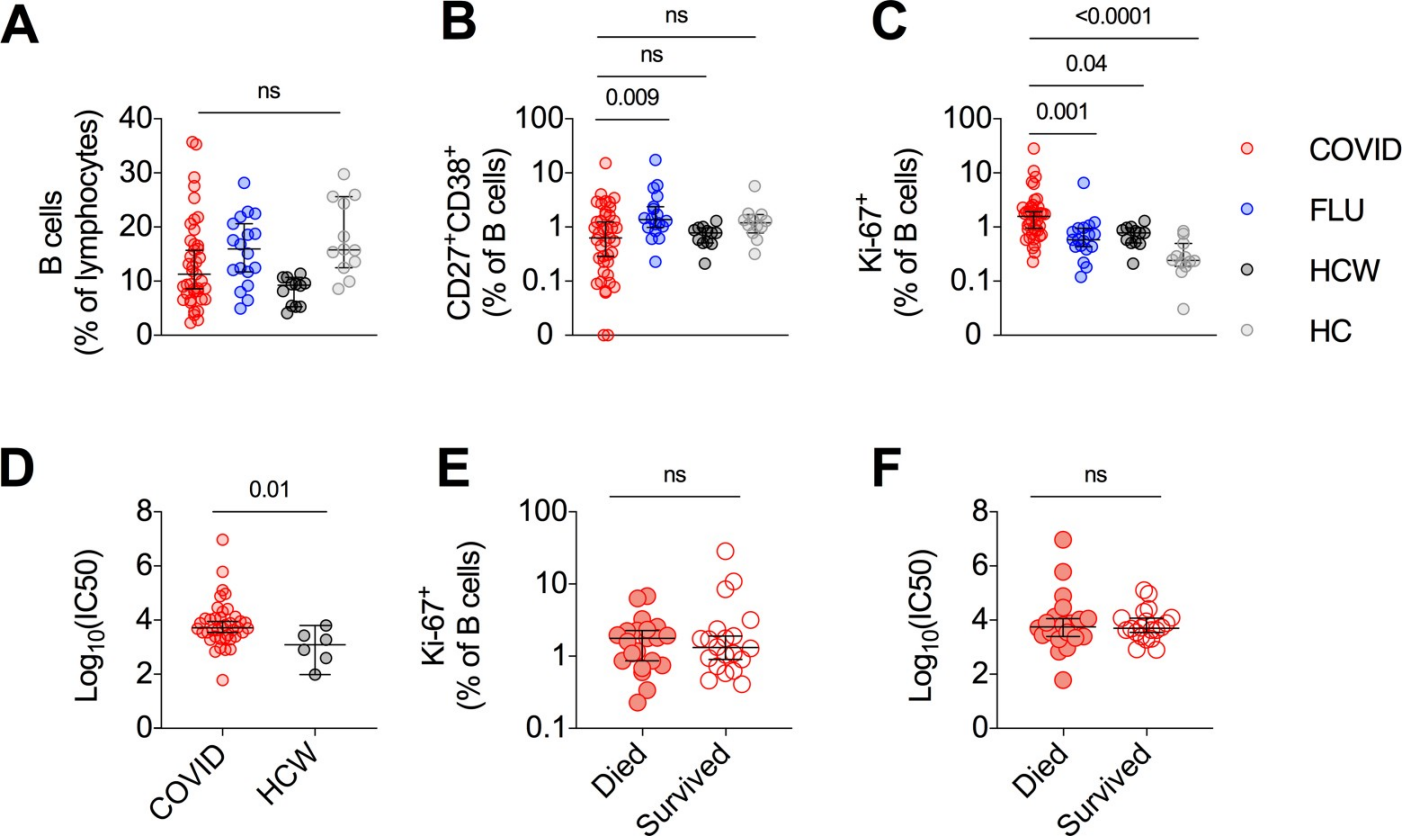
No association between SARS-CoV-2 neutralizing antibodies and fatal outcome in critically ill COVID-19 patients. **(A**-**C)** Frequency of B cells (A) CD27^+^CD38^+^ plasmablasts (B) and Ki-67^+^ proliferating B cells (C). **(D)** Log_10_ of the reciprocal plasma dilution that neutralized 50% of SARS-CoV-2 spike protein-expressing pseudovirus. 50% Inhibitory concentration (IC50). **(E**-**F)** Frequency of Ki-67^+^ B cells (E) and Log_10_ of the reciprocal plasma dilution that neutralized 50% of SARS-CoV-2 spike protein-expressing pseudovirus (F) with critically ill COVID-19 patients stratified by survival. Median ± 95% CI are shown. (A to C) Kruskal-Wallis tests with Dunn’s multiple comparison test. (D to F) Mann-Whitney U-test.

There was no significant alteration in B cell frequency in either the COVID cohort or FLU cohort as compared to HCWs or pre-pandemic HCs (Fig 2A). The frequency of CD27^+^CD38^+^ plasmablasts was highly variable in the COVID cohort with some individuals having large plasmablast responses (Fig 2B), but the overall frequency of plasmablasts was not significantly different from HCWs or HCs, and lower than in the FLU cohort. In contrast, the frequency of proliferating Ki-67^+^ B cells was significantly higher in the COVID cohort compared to all other groups (Fig 2C), consistent with a highly active B cell response. When SARS-CoV-2-specific neutralizing antibody responses were measured, patients in the critically ill COVID cohort had significantly higher titers than in convalescent individuals with mild disease (Fig 2D), as recently reported elsewhere [19]. However, we found no difference in the frequency of proliferating Ki-67^+^ B cells or the magnitude of neutralizing antibody responses and fatal outcomes in this cohort (Fig 2E and 2F).

### Association of serum protein concentrations with mortality in critically ill COVID-19 patients

**Fig 3:**
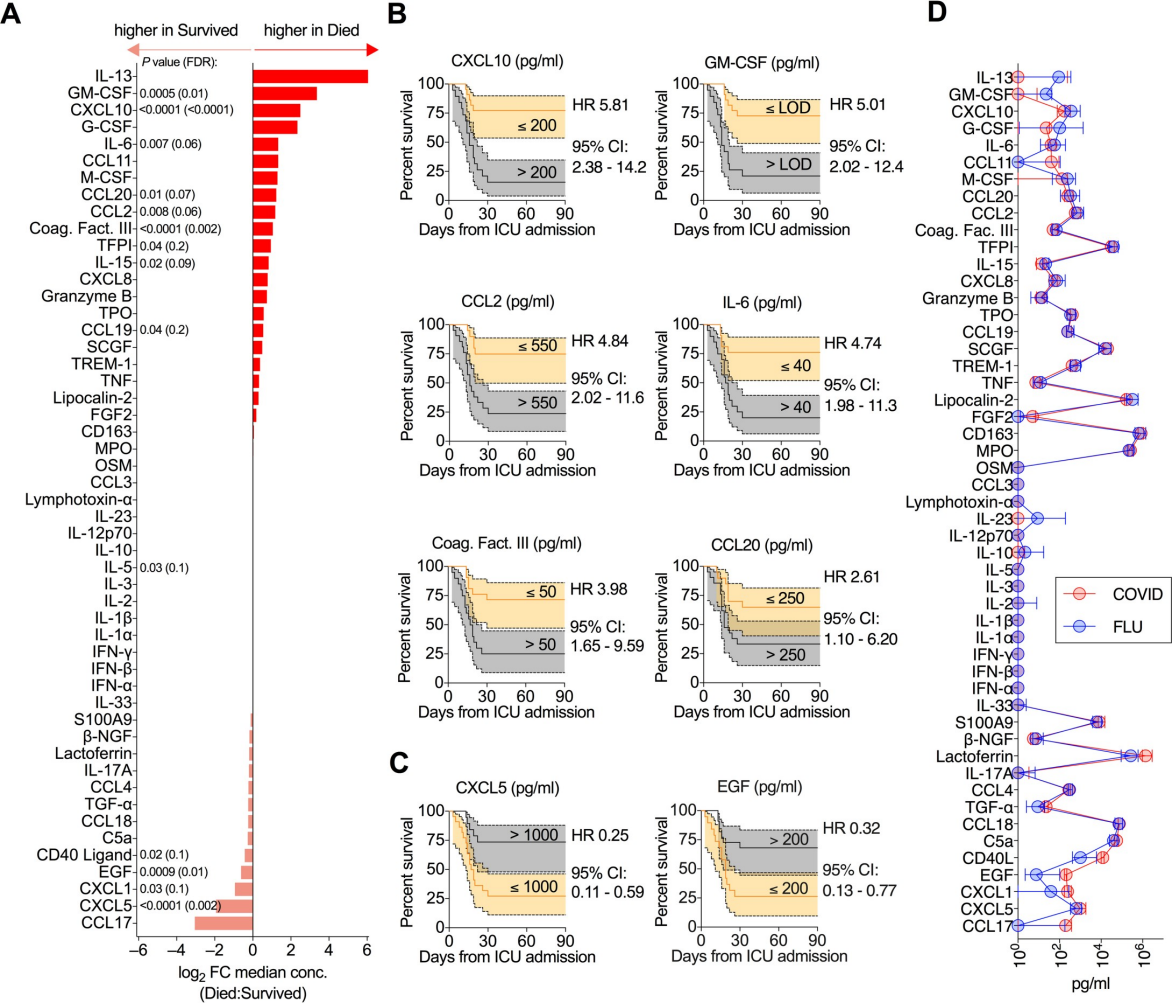
Mortality in critically ill COVID-19 patients is associated with perturbations in serum protein levels. **(A)** Fold-change in median serum protein concentration between critically ill COVID-19 patients who died versus survived. **(B**-**C)** Kaplan-Meier survival curves of critically ill COVID-19 patients based on concentration of the indicated analyte. Only proteins where FDR<0.1 (from (A)) are plotted. **(B)** Kaplan-Meier survival curves for serum proteins where above median expression is associated with increased mortality. **(C)** Kaplan-Meier survival curves for serum proteins where above median expression is associated with decreased mortality. **(D)** Median serum cytokine concentration in critically ill COVID-19 patients and critically ill influenza patients. (A) Mann-Whitney U-test with Benjamini-Hochberg FDR calculation on data presented in S4A Fig.

As disease severity and mortality have previously been associated with systemic alterations in serum cytokine and chemokine levels, we next examined these parameters in our cohort [3,44]. COVID patients who died had significantly elevated levels of a number of immune mediators including GM-CSF, CXCL10, IL-6, CCL20, CCL2, coagulation factor III, IL-15, TFPI (tissue factor pathway inhibitor), and CCL19 (Figs 3A and S3A). Above median expression of a number of these proteins was strongly associated with risk of death (Figs 3B and S3B). Elevated CXCL10 (HR = 5.81) and GM-CSF (HR = 5.01) were the strongest signals. A smaller fraction of serum immune mediators were inversely associated with death, including CD40 ligand, EGF, CXCL1, and CXCL5 (Figs 3A and S3A). The strongest negative association was with CXCL5 (HR = 0.25; Figs 3C and S3C). Perturbations in the serum concentration of many of the factors identified in this COVID cohort, in particular elevated CXCL10 and reduced CXCL5, have been previously linked to increased disease severity in other analyses [3,4]. Unexpectedly, median serum concentration of all cytokines/chemokines positively associated with mortality in the COVID cohort were even higher in the FLU cohort (Fig 3D), suggesting magnitude of the inflammatory response per se is not sufficient to explain the association of these proteins with mortality.

### Correlation analysis of mortality-associated immune parameters

**Fig 4:**
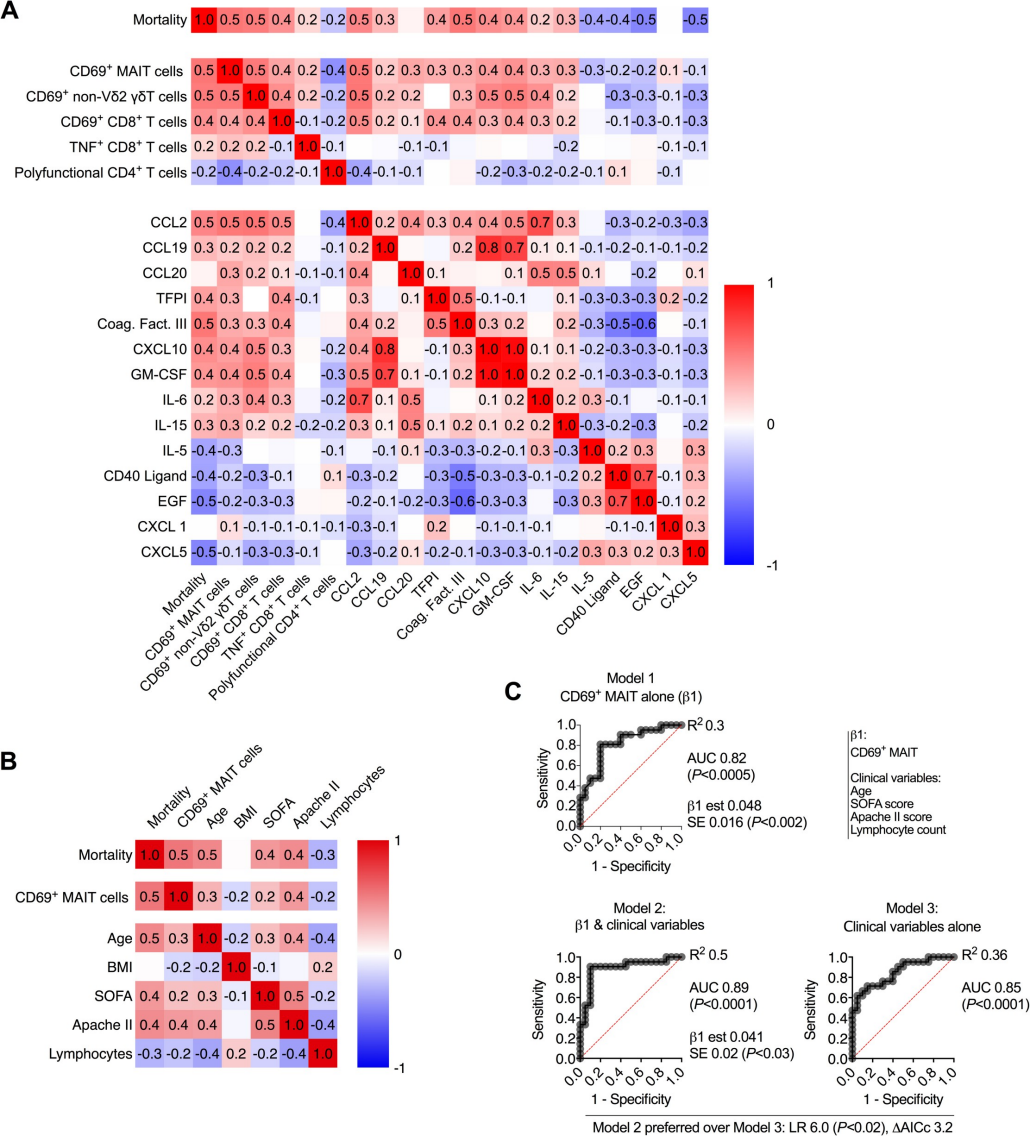
Activation of MAIT cells is associated with worse disease outcomes in critically ill COVID-19. **(A)** Pearson correlation of mortality with all statistically significant immunologic parameters. **(B)** Pearson correlation of mortality, MAIT cell CD69 expression, clinical measures, and timing of sampling. **(C)** ROC curve of MAIT cell CD69 expression (Model 1), ROC curve of MAIT cell CD69 expression combined with clinical variables (Model 2), and ROC curve of clinical variables alone (model 3). LR: likelihood ratio, ΔAICc: Difference in Akaike’s Information Criterion corrected.

We next investigated the interrelationship between mortality-associated immune parameters. MAIT cell CD69, non-Vδ2 γδT cell CD69, serum CCL2 concentration, and serum coagulation factor III concentration were the four parameters that had the greatest correlation with mortality (Pearson R = 0.5; Fig 4A). Conversely, serum EGF and CXCL5 concentrations were inversely correlated with mortality to a similar degree (R = −0.5; Fig 4A). Co-correlation of CD69 expression on T cell subsets had already been identified (S1D Fig), but we also determined that CD69 expression correlated with the elevated levels of mortality-associated serum proteins, and inversely correlated with survival-associated serum proteins (Fig 4A). Together these data suggest a concerted mortality-associated module of immune activation driven by inflammatory cytokines and chemokines; MAIT cell CD69 expression appears to be a sensitive marker of this process.

Antigen-specific T cell functions associated with differential mortality, such as CD8^+^ T cell TNF production and poly-functionality of CD4^+^ T cells, were only weakly correlated with this cytokine/chemokine/CD69 module (Fig 4A).

### Association between MAIT cell CD69 expression and clinical parameters of severity

A number of clinical parameters were also associated with increased mortality in the COVID cohort, including elevated Apache II and SOFA scores, increased age, and lymphopenia (S4A and S4B Fig), consistent with other reports [35,45–47]. The more pronounced lymphopenia in COVID patients who died manifested as a significant reduction in T cell counts, which was due to a proportional global reduction in abundance of all T cell populations analysed, as opposed to loss of any specific T cell subset (S4C Fig). BMI was not associated with mortality in this cohort (S4D Fig), in contrast to findings in other cohorts [48,49]. We investigated how MAIT cell CD69 expression, the mortality-associated immune marker with the greatest correlation, related to these clinical parameters. Unexpectedly, MAIT cell CD69 expression was more correlated with mortality than the well-established ICU admission Apache II or SOFA scores, and was equally correlated with mortality as age (Fig 4B).

To formally determine if measurement of MAIT cell CD69 was simply a proxy measure for one (or more) of these clinical parameters, we performed logistic regression analysis. ROC curve analysis demonstrated that MAIT cell CD69 expression alone was a reasonable predictor of mortality (Model 1, AUC = 0.82; Fig 4C). The model was further refined by addition of all identified mortality-associated clinical variables (Model 2, AUC = 0.89; Fig 4C). There was no suggestion of multicollinearity as for all variables, the variance inflation factor (VIF) was less than 1.7 (R^2^ <0.4). Furthermore, the model incorporating MAIT cell CD69 expression and all clinical parameters (Model 2) was preferred to a model comprising only clinical parameters (Model 3; Likelihood Ratio = 6.0 (*P*<0.02), difference in AICc = 3.2; Fig 4C). Thus, MAIT cell CD69 expression appears to be a robust and sensitive independent correlate of differential survival in critically ill COVID-19 patients.

### Association of immune parameters and disease severity in critically ill ICU influenza patients

To determine if the mortality-associated immune parameters identified in COVID-19 were disease-specific, we queried them against our FLU cohort. ECMO (extracorporeal membrane oxygenation) treatment is the standard of care for the most critically ill influenza patients not responding to standard mechanical ventilation, and effectively reduces mortality [50]. As there was only a single death in this cohort, we employed ECMO treatment as a clinically-defined indicator of disease severity. Thus, we stratified the FLU cohort based on those who received ECMO treatment or died, and those who did not, resulting in nine individuals in each group overall (Table 1).

**Fig 5:**
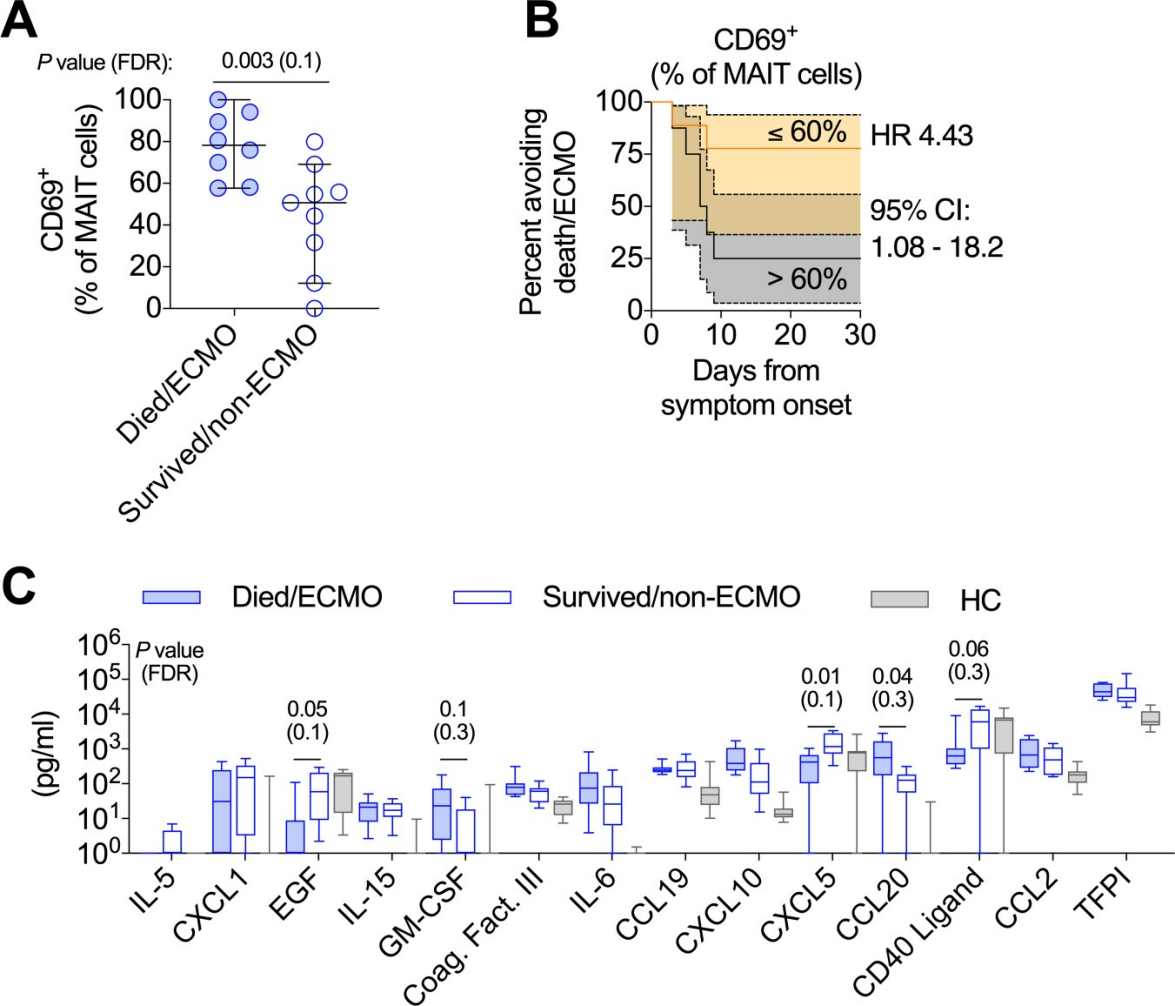
Activation of MAIT cells is associated with worse disease outcomes in critically ill influenza patients. Investigation of immunologic measures that were associated with mortality in the critically ill COVID-19 cohort, in a cohort of critically ill influenza patients. **(A)** MAIT cell CD69 expression on critically ill influenza patients who died or required ECMO versus those who did not. **(B)** Kaplan-Meier survival curve of disease outcome of critically ill influenza patients based on above or below median MAIT cell CD69 expression. **(C)** Examination of the concentration of 14 serum proteins in the critically ill influenza cohort and healthy controls, which were associated with mortality in the critically ill COVID-19 cohort (Fig 3). Dots represent individual patients (A), and median ± 95% CI are shown. (C) Median, IQR, and min to max are shown. (A and C) Mann-Whitney U-test with Benjamini-Hochberg FDR calculation.

Examination of general activation of T cells (by CD69), as well as cytokine production by antigen-specific T cell responses (stimulation with NP+M1 overlapping peptide pool) identified elevated activation of MAIT cells as the only T cell parameter differentially associated with disease severity in this cohort (Figs 5A and S5). Stratification of the cohort by median CD69 expression in MAIT cells was associated with disease severity (death/ECMO; HR = 4.43; Fig 5B), similar to the COVID cohort. A number of the mortality-associated serum proteins in the COVID cohort were also associated with differential disease severity in the FLU cohort. Serum EGF and CXCL5 concentrations were significantly higher in surviving non-ECMO patients (Fig 5C). This differential expression of EGF actually reflected a loss in EGF expression in patients with more severe disease as compared to milder disease and healthy controls. This severity-associated decrease in serum protein concentration was also observed for soluble CD40L (Fig 5C). Conversely, GM-CSF and CCL20 were elevated in the more severe patients, and CXLC10 trended in this direction as well (*P* = 0.06; Fig 5C). Interestingly, coagulation factor III and TFPI (also part of the coagulation pathway) were not different between the two groups, consistent with the observation that coagulopathy is a more prominent feature of severe COVID-19 than severe influenza infection [51].

### Identification of signalling pathways associated with mortality in critically ill COVID-19 patients by single-cell RNA-sequencing

To further investigate the phenotypes observed within the cellular and proteomic analysis, we conducted single-cell RNA sequencing (scRNA-seq) on PBMCs from a subset of patients in our COVID (*n* = 12) and FLU (*n* = 12) cohorts (S3 Table). In addition, using Seurat [52], we integrated these samples with a prior scRNA-seq dataset [24], which used the same library preparation technology, of six additional critically ill COVID-19 patients and six healthy donors to yield an integrated dataset with >75,000 cells from 36 individuals: 18 COVID-19 patients (COVIDseq cohort; *n* = 11 died and *n* = 7 survived), 12 influenza patients (FLUseq cohort; *n* = 6 ECMO and *n* = 6 non-ECMO), and 6 healthy controls (S3 Table).

**Fig 6:**
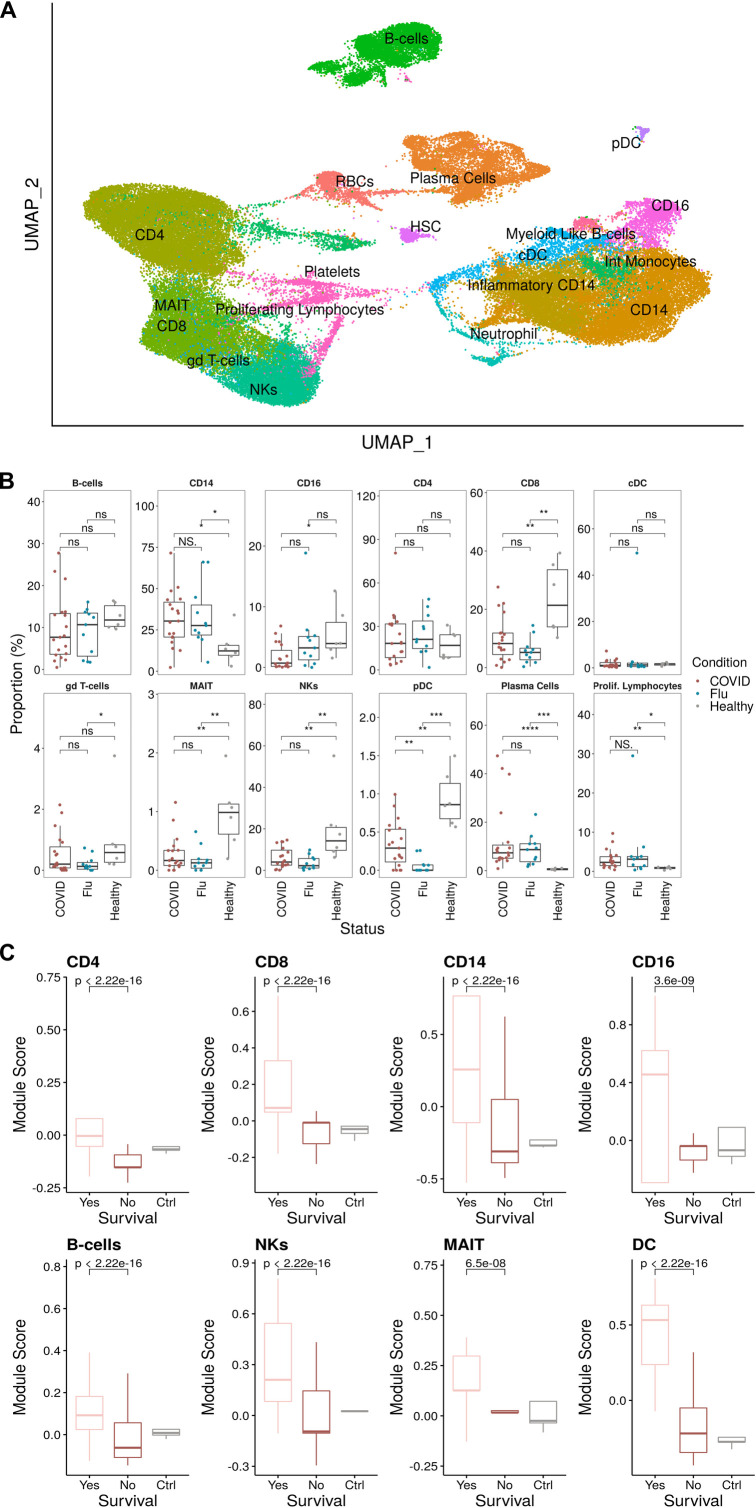
Elevated type I interferon signaling across multiple cell populations in critically ill COVID-19 patients who survive. **(A)** UMAP projection of the scRNA-seq cohort consisting of 75,601 PBMCs (43,687 cells from COVID, 16,616 Healthy, and 15,298 Flu) colored by manually annotated cell types. **(B)** Relative cell proportions within each individual separated by disease condition. Statistical tests were conducted using the Wilcoxon rank-sum test between each condition. Summary values were subsequently displayed using the boxplot. The box is equivalent to the interquartile range (IQR) with the median as the center, and whiskers correspond to the 25^th^ percentile—1.5x IQR or the lowest value, and 75^th^ percentile +1.5x IQR or the highest value. **(C)** Type I IFN module scores for each major cell type compared between COVID survival and death with healthy controls as a reference. Significance was determined using the Wilcoxon test.

We then computed cell clusters using a graph-based clustering method, visualized using uniform manifold approximation and projection (UMAP), and performed manual annotations based on known distinguishing markers (Figs 6A and S6, and S4 and S5 Tables). From this, we observed depletion of NK cells, CD8^+^ T cells, CD16^+^ monocytes, and plasmacytoid dendritic cells within the COVIDseq cohort as compared to healthy controls (Fig 6B), consistent with a prior report using the same Wilcoxon-based analysis method [53]. As expected, plasma cell frequency was increased in both the COVIDseq and FLUseq cohorts compared to healthy controls (Fig 6B). However, using a Dirichlet multinomial regression model, which accounts for the abundance of all cell types [29,54], there were no significant alterations in cellular proportions in the COVIDseq cohort (S7 Fig), suggesting that prior reports of preferential cell loss may be contingent on the analysis method used.

There were no significant differences in the frequency of any cell subsets in patients in the COVIDseq cohort who died versus those who survived (S8 Fig). However, elevated *CD69* expression in MAIT cells was observed in patients in the COVIDseq cohort who died (S9 Fig), consistent with the protein-level analysis (Fig 1E).

Type I IFN dysregulation has been commonly described in association with severe COVID-19 disease [5,6]. To investigate whether this observation applied to survival, we scored patients who died (*n* = 11) versus survived (*n* = 7) from the COVIDseq cohort on expression of curated type I IFN associated genes from the REACTOME database [55], using healthy controls as the reference. In patients who survived, there were significantly elevated type I IFN module scores in all major cell types (Figs 6C and S10).

**Fig 7:**
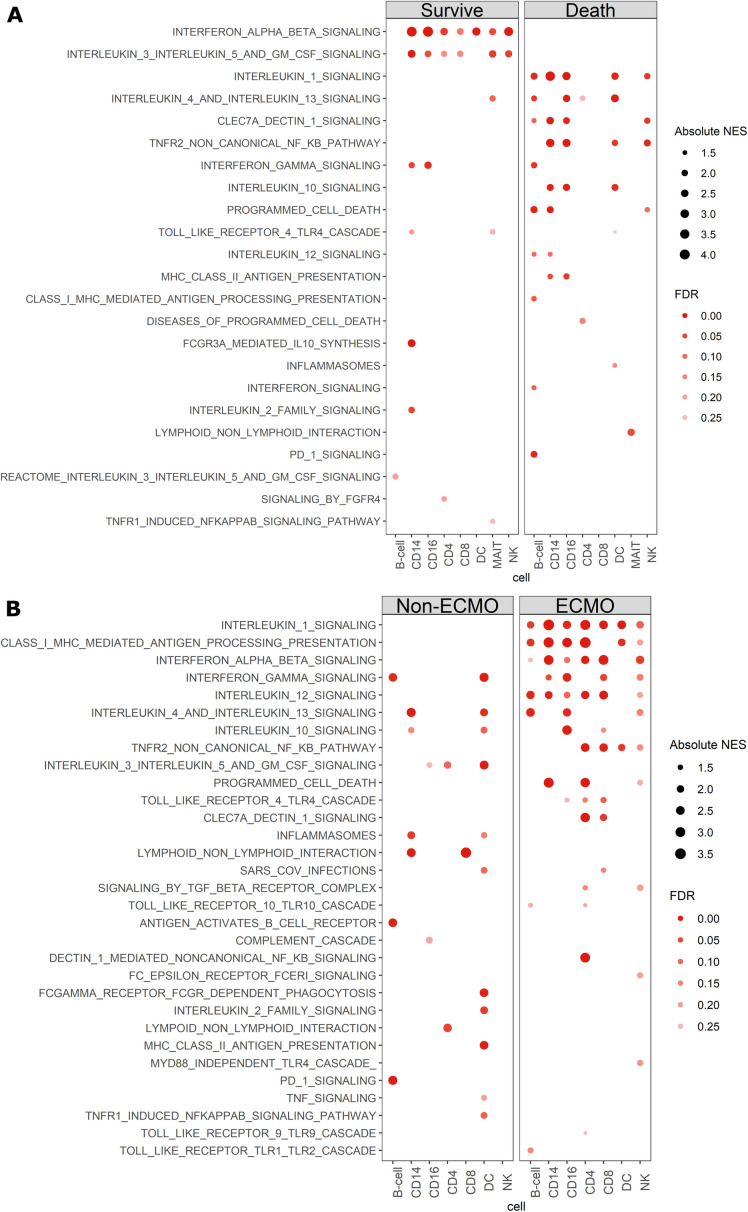
Divergent cytokine signaling pathways associated with clinical outcome in critically ill COVID-19 and influenza patients. **(A,B)** Curated dotplot of enriched pathways between both survival and death in critically ill COVID patients (A) and critically ill influenza patients (B). Enriched pathways were obtained via reactome pathways identified via GSEA between survival and death conditions within the COVID dataset, or ECMO non-ECMO in the influenza cohort. FDRs were calculated based on q-values obtained from the hypergeometric test applied to the geneset followed by multiple hypothesis correction using the Benjamini-Hochberg method. NES: normalized enrichment score, FDR: false discovery rate.

We next sought to determine which signalling pathways might be differentially stimulated in patients who died versus survived in the COVIDseq cohort, using a generalized linear model (see methods). Patients who survived demonstrated a very focused immune response with consistent upregulation of type I IFN signalling across multiple cell types, and a lack of consistent enrichment of other major signalling pathways (Fig 7A). In contrast, in patients who died, type I IFN signalling was not enriched in any cell population and instead broad pro- and anti-inflammatory processes were simultaneously observed (S6 Table). For example, the IL-10 signalling pathway was enriched in CD14^+^ monocytes, CD16^+^ monocytes, CD8^+^ T cells, and dendritic cells, while inflammatory responses (IL-1, IFNγ, and TNF signalling) were enriched in CD14^+^, CD16^+^ monocytes, NK cells, B cells, dendritic cells and MAIT cells. Thus, broad, un-focused immune responses appear to be a hallmark of fatal COVID-19 disease.

We investigated whether these skewed responses were also observed in the context of severe influenza infection. Consistent with the data from the COVIDseq cohort, ECMO patients (*n* = 6) in the FLUseq cohort had elevated signals for multiple inflammatory pathways across several cell types (Fig 7B). However, in contrast to the COVIDseq cohort, type I IFN signalling pathways were significantly enriched in B cells, monocytes (CD14^+^ and CD16^+^), T cells (CD4^+^ and CD8^+^), and NK cells of the ECMO patients in the FLUseq cohort. Taken together, these data suggest that focused elevated IFN signalling is likely a key factor in increased immune cell activation in severe influenza infection, while in contrast other cytokines are key for the activated phenotype of MAIT cells (and other T cell subsets) in severe COVID-19.

Finally, we further investigated the differences in signalling pathways between COVID-19 and influenza in critically ill patients by performing a head-to-head comparison between the two disease cohorts stratified by disease severity (survived COVID-19 versus non-ECMO, and died COVID-19 versus ECMO influenza). This comparison revealed a striking enrichment in interferon signalling across all cell types in the COVIDseq patients who survived as compared to the FLUseq patients who did not require ECMO (S11A Fig), further reinforcing the importance of these pathways in COVID-19 survival (Fig 7A). Comparison of COVIDseq patients who died to FLUseq patients who required ECMO resulted in a less clear demarcation (S11B Fig). with notable differences being multiple elevated B cell pathways, and broad differences in pathways enriched in monocytes and DCs of COVIDseq patients who died compared to FLUseq patients who required ECMO. These data provide potential avenues for further comparison of the differential biology of these two diseases.

## Discussion

The overall aim of this study was to investigate the association between SARS-CoV-2-specific T cell responses, neutralizing antibody titers, and fatal outcome in critical COVID-19. Our key finding was that amongst T cells, activation of MAIT cells was the strongest predictor of a fatal outcome. By contrast, only very modest associations between alterations in antigen-specific T cell responses and fatal outcomes were observed. Magnitude of the SARS-CoV-2-specific neutralizing antibody response was not associated with disease outcome.

A number of prior studies have linked perturbed SARS-CoV-2-specific T cell and antibody responses with disease severity, including death [15,16,19,21,56]. However, many of these studies compared survival across the entire cohort studied, which necessarily results in the “survived” group including patients with heterogeneous phenotypes, including mild disease. Thus it is often difficult to directly assess if parameters identified as associated with disease severity can also accurately differentiate mortality versus survival for critically ill patients. In this study we examined only critically ill patients, allowing us to identify immune parameters specifically associated with survival in a more clinically homogenous population. Thus the lack of strong associations between conventional spike-specific T cell functions and neutralizing antibody titers and mortality suggests that neither excessive nor defective antigen-specific responses likely contribute to mortality in critical COVID-19 infection.

In contrast, altered cytokine/chemokine levels (e.g. CXCL10, GM-CSF, CCL2, and IL-6) were strongly associated with increased mortality in our cohort. These findings validate prior reports that elevated cytokine levels, particularly CXCL10 (IP-10), as associated with both increased severity and mortality [2,3,57]. Furthermore, the observation of increased IL-6 levels in fatal disease is highly consistent with the clinical benefit seen with Tocilizumab (anti-IL-6 receptor) treatment reported in the recently-reported RECOVERY trial [58]. Thus, an association between systemic inflammation and increased mortality appears to be reproducible across multiple cohorts.

While the MAIT cell T cell receptor recognizes bacterial- and yeast-derived vitamin B2 metabolites, these cells can also respond to viral infections via cytokines [59]. Such cytokines are most active in combination and include IL-12, IL-18, and IFNα, with a critical role for TNF in vivo [60–62]. MAIT cell activation was strongly correlated with mortality-associated cytokine and chemokine levels. Moreover, MAIT cell activation was more associated with fatal outcomes than any other parameter measured, including the clinical prognostic scores SOFA and APACHE II. MAIT cell activation in the FLU cohort was also strongly associated with disease severity. Thus, MAIT cell CD69 expression appears to be a highly sensitive, though not necessarily disease-specific, marker of disease severity in severe viral pneumonia, likely through integration and amplification of multiple cytokine-driven signals.

Several studies have specifically investigated MAIT cells in the context of COVID-19 [37,38,63]. MAIT cells were highly activated, and the degree of activation positively correlated with disease severity in two of the studies [37,63]. These findings are highly consistent with our findings of an association between elevated MAIT cell CD69 expression and mortality. Flament and colleagues [63] reported similar findings in a cohort comprising both moderate and severe COVID-19. One limitation of this previous study was it focused solely on innate/innate-like lymphocyte populations, making it challenging to contextualize these findings within the overall immune response. Additionally, this study did not investigate other viral pneumonias, so the specificity of MAIT cell activation to COVID-19 could not be assessed. Using a broad experimental approach in the current study allowed us to determine the importance of MAIT cell activation as an immune correlate of mortality in COVID-19. Thus, our current work and this prior study serve as robust independent validations of this central finding. The authors also identified a shift away from type I IFN-driven signals being associated with increased MAIT cell activation and disease severity, which is highly consistent with our transcriptomic analysis.

Overall, the in vivo function of such activated MAIT cells remains to be determined. Although impacts could include cytotoxicity [63] and cytokine/chemokine secretion [61,64], given the relatively low frequencies of these cells in severe disease, an “amplifier” role in inflammatory disease through activation and recruitment to tissues seems likely [65,66]. However, in this context they could also represent a very sensitive biomarker of an activation process which affects many T cell subsets that are more numerous and more directly pathogenic upon activation.

In both COVID-19 and influenza we observed upregulation of a number of inflammatory pathways (at the transcriptional and serum protein levels) in patients with the most extreme clinical manifestations. However, the role of type I IFNs was divergent between COVID-19 and influenza, consistent with reports of differential induction in these two viral infections [53,67]. Elevated type I IFN signalling was associated both with survival in critical COVID-19 patients and also disease severity in ICU influenza patients. These data are consistent with recent reports that have demonstrated anti-IFN auto-antibodies and genetic defects of type I IFN immunity in a sizeable fraction of severe COVID-19 patients [7–9]. Collectively, these data lend further support to the hypothesis that an impaired type I IFN response is a key process in COVID-19 pathogenesis [5,6]. Our data argue against the notion that a hallmark of COVID-19 is an overwhelming “cytokine storm”, a model that has recently been questioned [44,68,69]. Instead, a more complex and nuanced dysregulation of acute responses appears to be associated with fatal outcomes.

Comparison of critically ill COVID-19 and influenza patients identified several additional interesting differences between the two diseases. Elevated soluble CD40L and EGF were associated with survival in both cohorts (Figs 3A and 5C), but the underlying reasons appear different. Serum EGF and CD40L levels were elevated in COVID-19 patients who survived compared to patients who died or healthy controls. By contrast, in influenza infection, serum EGF and CD40L levels were actually decreased below baseline in patients with more severe disease. A prior report found a similar association with CD40L [70]. Activated platelets are a major source of serum CD40L [71], so these data may be functionally relevant to the thrombotic events seen in COVID-19 (as compared to influenza) [72]. The EGF receptor (EGFR) is an entry receptor for influenza virus [73]. Theoretically, a specific decrease in EGF might allow for increased binding of virions to EGFR, a mechanistic and testable explanation for this association. Lastly, the direct single-cell RNA-sequencing comparison of COVID-19 and influenza patients with the most severe disease revealed a number of immune pathways in B cells, monocytes, and DCs that were differential between the two infections, potentially relevant to the auto-antibody generation seen in severe COVID-19 infection [74]. The latter findings warrant more in-depth investigation in larger patient cohorts, but illustrate the strength of a direct comparison between two critically ill cohorts with virus-induced ARDS, with the potential to yield novel insights into immunopathogenesis.

There are two specific limitations to our current study. One, all work was performed on peripheral blood, so further studies will be required to confirm these findings are translatable to the lung. Two, the study is cross-sectional in nature, so further work will be required to understand how these mortality-associated immune parameters develop over the course of infection.

In conclusion, our comprehensive immunophenotyping study yields an enhanced understanding of the differential immunopathogenic processes driving critical COVID-19 and influenza, which can translate into improved immunotherapeutic approaches in patients with severe viral pneumonitis. This combined with the identification of a sensitive and simple immune correlate of disease severity (MAIT cell activation) can ultimately improve patient risk stratification.

## Supporting information

S1 Fig Additional data related to Fig 1. **(A)** Frequency of T cells as a fraction of lymphocytes. **(B**-**C)** Fraction of CD4^+^ and CD8^+^ conventional T cells (B) and unconventional T cell populations (C) within the CD3^+^ T cell population. **(D)** Pearson correlation in CD69 expression between each of the indicated T cell populations. **(E**-**F)** Measures of conventional CD4^+^ and CD8^+^ T cell activation by Ki-67 expression (E) or co-expression of HLA-DR and CD38 (F). **(G**-**H**) Frequency of spike-specific CD4^+^ T cell and CD8^+^ T cells producing IL-17A (G) and IL-4 (H). Dashed line indicates the upper 95% confidence interval for responses detected in pre-pandemic healthy controls. **(I)** Polyfunctionality (CD107a, IFNγ, TNF, and/or IL-2) of spike-specific CD4^+^ T cells (top) and CD8^+^ T cells (bottom) between critically ill COVID-19 patients and convalescent health care workers (HCW). Dots represent individual patients. Median ± 95% CI are shown. (A to C, E, and F) Kruskal-Wallis tests with Dunn’s multiple comparison test. (G to I) Mann-Whitney U-test.
(TIFF)

S2 Fig Additional data related to Fig 1. **(A**-**B)** Median expression of the indicated cytokine or activation marker in CD4^+^ T cells (A) or CD8^+^ T cells (B) of critically ill COVID-19 patients who died or survived. **(C)** Kaplan-Meier survival curve of critically ill COVID-19 patients based on fraction of TNF-producing, spike-specific CD8^+^ T cells. **(D)** Fraction of spike-specific CD8^+^ T cells that are polyfunctional (≥2 cytokines produced) in critically ill COVID-19 patients that died versus survived. **(E)** Proportion of spike-specific CD4^+^ T cells that produce 2, 3, or 4 cytokines (of CD107a, IFNγ, TNF, or IL-2) in critically ill COVID-19 patients who died or survived. **(F)** Proportion of spike-specific CD4^+^ T cells that produce each of the indicated combinations of cytokines in critically ill COVID-19 patients who died or survived. **(G)** CD45RA and CCR7 expression on spike-specific CD4^+^ T cells from critically ill COVID-19 patients who died or survived. Dots represent individual patients. Median ± 95% CI are shown. (B) Kruskal-Wallis tests with Dunn’s multiple comparison test. (A, B, D, E, and G) Mann-Whitney U-test with Benjamini-Hochberg FDR calculation. (F) Mann-Whitney U-test.
(TIFF)

S3 Fig Additional data related to Fig 3. **(A)** Serum concentration of 51 protein analytes. Ordered as in Fig 3A. Median ± 95% CI are shown. **(B)** Kaplan-Meier survival curves for serum proteins where above median expression is associated with increased mortality. **(C)** Kaplan-Meier survival curves for serum proteins were above median expression is associated with decreased mortality.
(TIFF)

S4 Fig Additional data related to Fig 4. **(A)** Comparison of clinical measures between critically ill COVID-19 patients who died or survived. **(B)** Kaplan-Meier survival curves for clinical parameters from panel (A). **(C)** Absolute counts (per L) of the indicated lymphocyte population in critically ill COVID-19 patients who died or survived. **(D)** Comparison of body-mass index (BMI) between critically ill COVID-19 patients who died or survived (left), and Kaplan-Meier survival curve (right). Dots represent individual patients. Median ± 95% CI are shown. (A and D) Mann-Whitney U-test with Benjamini-Hochberg FDR calculation; (C) Two-way ANOVA with Sidak’s multiple comparison test.
(TIFF)

S5 Fig Additional data related to Fig 5. **(A**-**C)** Median expression of CD69 on the indicated T cell population (A), or the indicated cytokine or activation marker in CD4^+^ T cells (B) or CD8^+^ T cells (C) of critically ill influenza patients who died or required ECMO treatment versus those who did not. **(D**-**E)** Fraction of NP+M1-specific CD4^+^ T cells (D) CD8^+^ T cells (E) that are polyfunctional (≥2 cytokines produced) in critically ill influenza patients who died or required ECMO treatment versus those who did not. **(F)** Proportion of NP+M1-specific CD4^+^ T cells that produce 2, 3, or 4 cytokines (of CD107a, IFNγ, TNF, or IL-2) in critically ill influenza patients who died or required ECMO treatment versus those who did not. **(G)** Proportion of spike-specific CD4^+^ T cells that produce each of the indicated combinations of cytokines in critically ill influenza patients who died or required ECMO treatment versus those who did not. Dots represent individual patients. Median ± 95% CI are shown. (A to F) Mann-Whitney U-test with Benjamini-Hochberg FDR calculation.
(TIFF)

S6 Fig Additional data related to Fig 6. Top 3 genes for each cluster (used to annotate cell populations in Fig 6A).
(TIFF)

S7 Fig Additional data related to Fig 6. Relative cell proportions within each individual separated by disease condition. Statistical tests were conducted using Dirichlet regression between each condition. Mean ± standard deviation are shown.
(TIFF)

S8 Fig Additional data related to Fig 6. **(A)** Relative cell proportions within each COVID individual separated by survival and death. **(B)** Relative cell proportions within each influenza individual separated by ECMO and non-ECMO. Statistical tests were conducted using the Wilcoxon rank-sum test between each condition. Summary values were subsequently displayed using the boxplot. The box is equivalent to the interquartile range (IQR) with the median as the center, and whiskers correspond to the 25^th^ percentile—1.5x IQR or the lowest value, and 75^th^ percentile +1.5x IQR or the highest value.
(TIFF)

S9 Fig Additional data related to Fig 6. Expression of CD69 in survived and died COVID-19 patients. Significance was determined using the Wilcoxon test.
(TIFF)

S10 Fig Additional data related to Figs 6 and 7. Expression pattern of the individual genes across the cell subsets and disease cohorts within the IFN gene module from Fig 6C.
(TIFF)

S11 Fig Additional data related to Fig 7. Curated dotplot of enriched pathways between (A) survived critically ill COVID patients versus survived/non-ECMO critically ill influenza patients and (B) died critically ill COVID patients versus died/ECMO critically ill influenza patients. Enriched pathways were obtained via reactome pathways identified via GSEA. FDRs were calculated based on q-values obtained from the hypergeometric test applied to the geneset followed by multiple hypothesis correction using the Benjamini-Hochberg method. NES: normalized enrichment score, FDR: false discovery rate.
(TIFF)

S1 Table Antibodies for flow cytometry. (XLSX)

S2 Table Antibodies for intracellular cytokine staining. (XLSX)

S3 Table Clinical metadata for the scRNA-seq dataset. (XLSX)

S4 Table Cell counts for each cell type. (XLSX)

S5 Table Full gene list for annotating cell types. (XLSX)

S6 Table Differentially expressed genes per cell type by disease severity. (XLSX)
