# Supplementary figures and images for: Identification of immune correlates of fatal outcomes in critically ill COVID-19 patients
Source: PLoS Pathog. 2021 Sep 16;17(9):e1009804. doi: 10.1371/journal.ppat.1009804 (PMC8445447; doi:10.1371/journal.ppat.1009804)

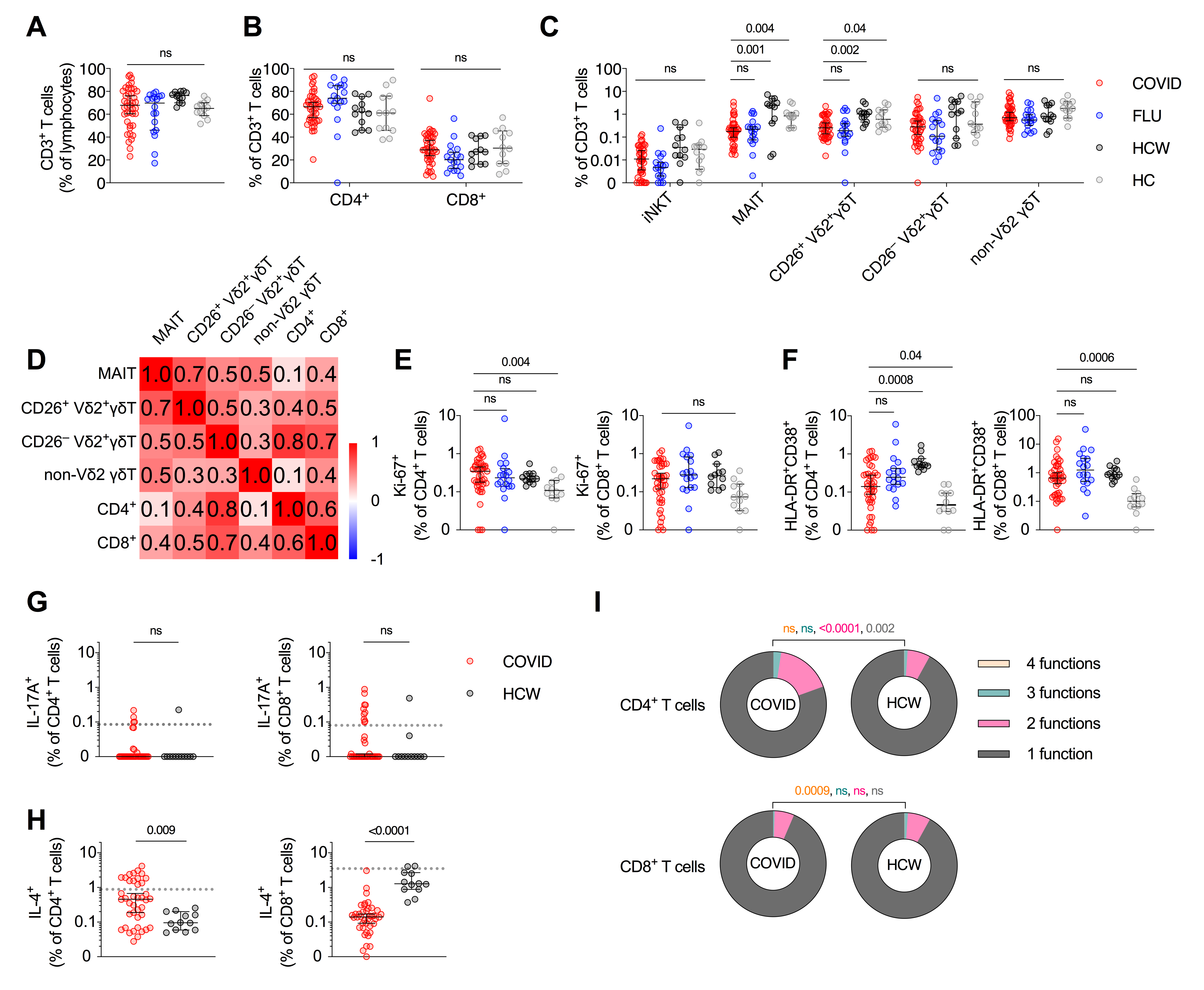

Supplement: S1 Fig — (A) Frequency of T cells as a fraction of lymphocytes. (B-C) Fraction of CD4+ and CD8+ conventional T cells (B) and unconventional T cell populations (C) within the CD3+ T cell population. (D) Pearson correlation in CD69 expression between each of the indicated T cell populations. (E-F) Measures of conventional CD4+ and CD8+ T cell activation by Ki-67 expression (E) or co-expression of HLA-DR and CD38 (F). (G-H) Frequency of spike-specific CD4+ T cell and CD8+ T cells producing IL-17A (G) and IL-4 (H). Dashed line indicates the upper 95% confidence interval for responses detected in pre-pandemic healthy controls. (I) Polyfunctionality (CD107a, IFNγ, TNF, and/or IL-2) of spike-specific CD4+ T cells (top) and CD8+ T cells (bottom) between critically ill COVID-19 patients and convalescent health care workers (HCW). Dots represent individual patients. Median ± 95% CI are shown. (A to C, E, and F) Kruskal-Wallis tests with Dunn’s multiple comparison test. (G to I) Mann-Whitney U-test. (TIFF) [file ppat.1009804.s001.tiff]

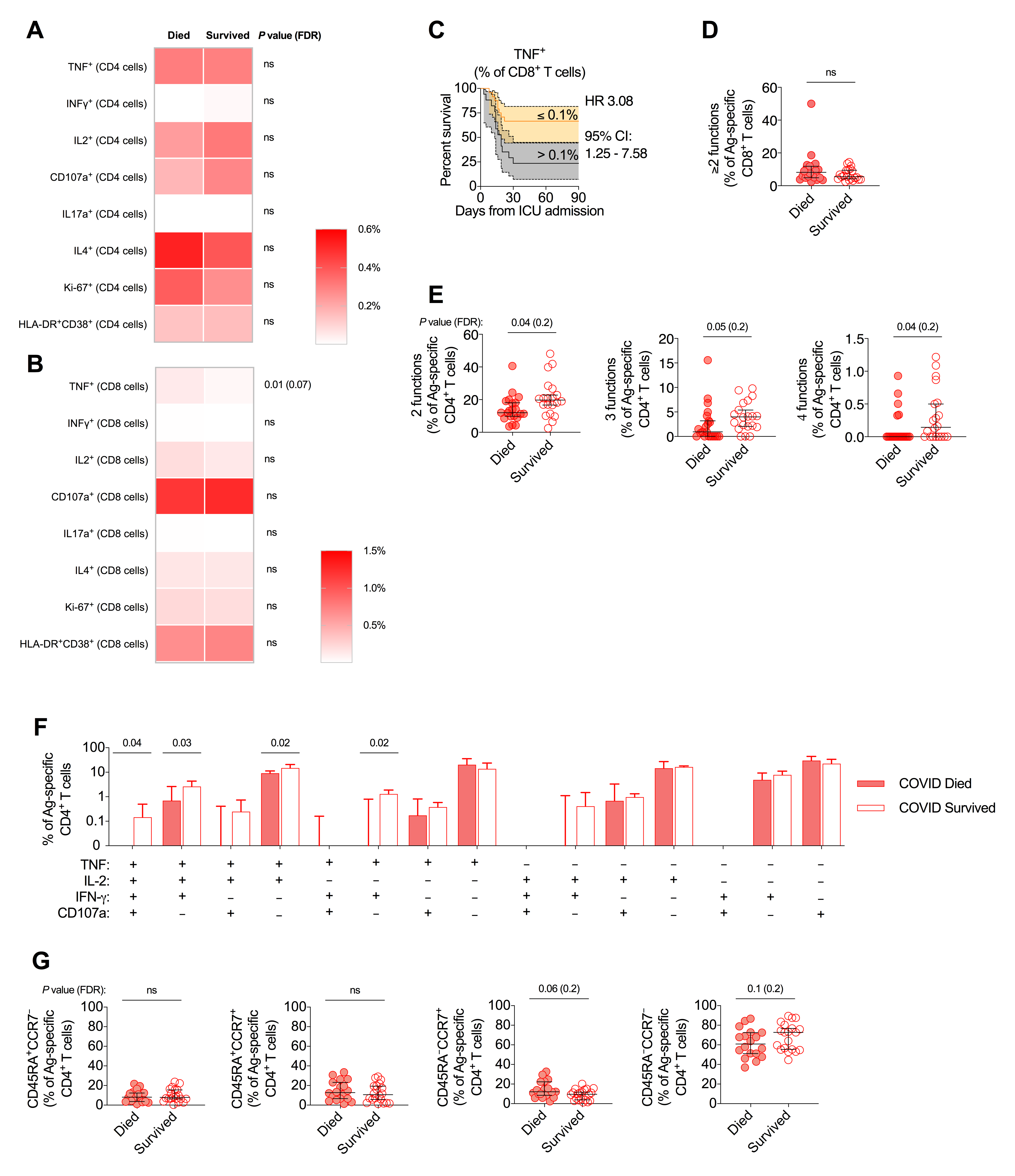

Supplement: S2 Fig — (A-B) Median expression of the indicated cytokine or activation marker in CD4+ T cells (A) or CD8+ T cells (B) of critically ill COVID-19 patients who died or survived. (C) Kaplan-Meier survival curve of critically ill COVID-19 patients based on fraction of TNF-producing, spike-specific CD8+ T cells. (D) Fraction of spike-specific CD8+ T cells that are polyfunctional (≥2 cytokines produced) in critically ill COVID-19 patients that died versus survived. (E) Proportion of spike-specific CD4+ T cells that produce 2, 3, or 4 cytokines (of CD107a, IFNγ, TNF, or IL-2) in critically ill COVID-19 patients who died or survived. (F) Proportion of spike-specific CD4+ T cells that produce each of the indicated combinations of cytokines in critically ill COVID-19 patients who died or survived. (G) CD45RA and CCR7 expression on spike-specific CD4+ T cells from critically ill COVID-19 patients who died or survived. Dots represent individual patients. Median ± 95% CI are shown. (B) Kruskal-Wallis tests with Dunn’s multiple comparison test. (A, B, D, E, and G) Mann-Whitney U-test with Benjamini-Hochberg FDR calculation. (F) Mann-Whitney U-test. (TIFF) [file ppat.1009804.s002.tiff]

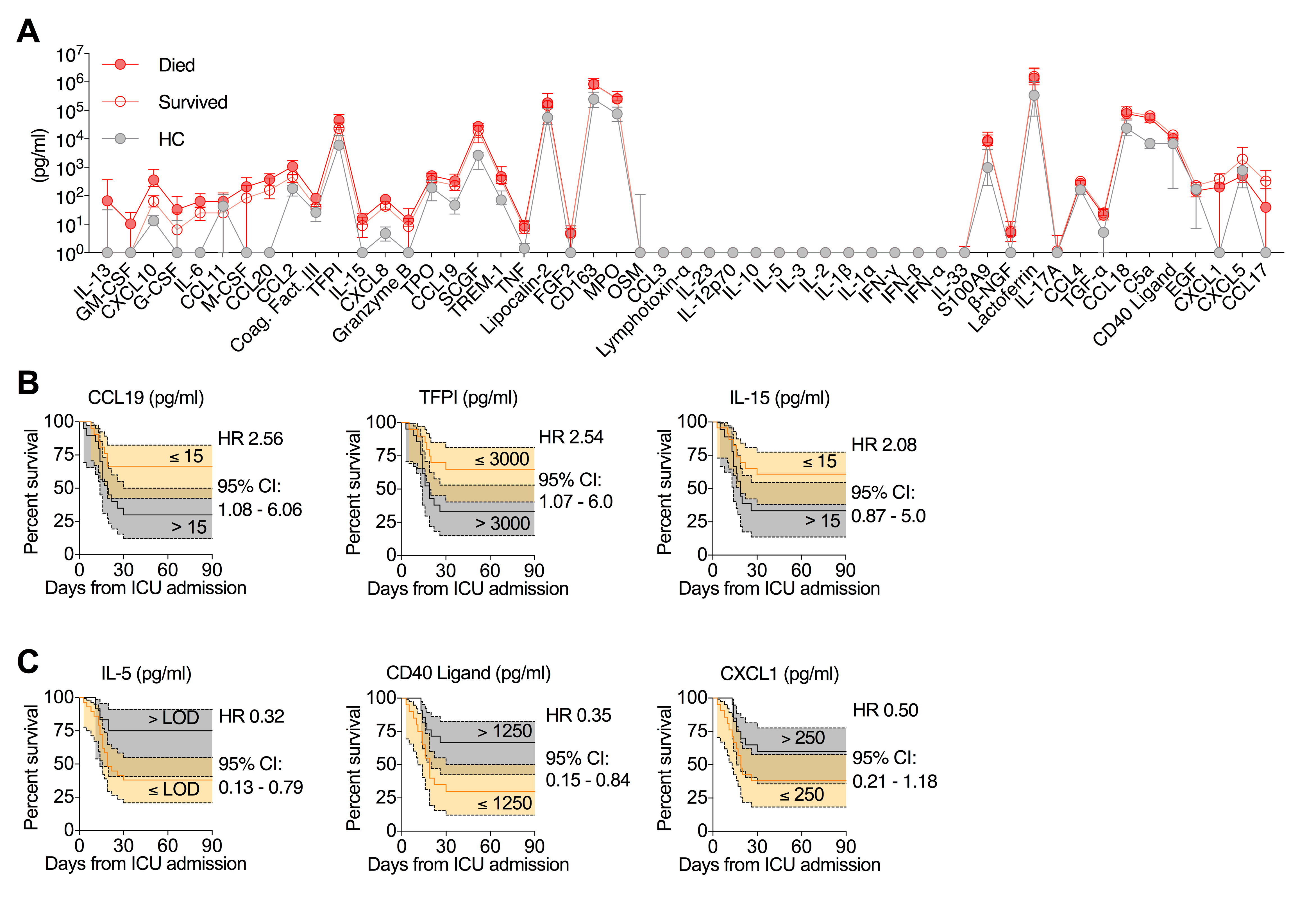

Supplement: S3 Fig — (A) Serum concentration of 51 protein analytes. Ordered as in Fig 3A. Median ± 95% CI are shown. (B) Kaplan-Meier survival curves for serum proteins where above median expression is associated with increased mortality. (C) Kaplan-Meier survival curves for serum proteins were above median expression is associated with decreased mortality. (TIFF) [file ppat.1009804.s003.tiff]

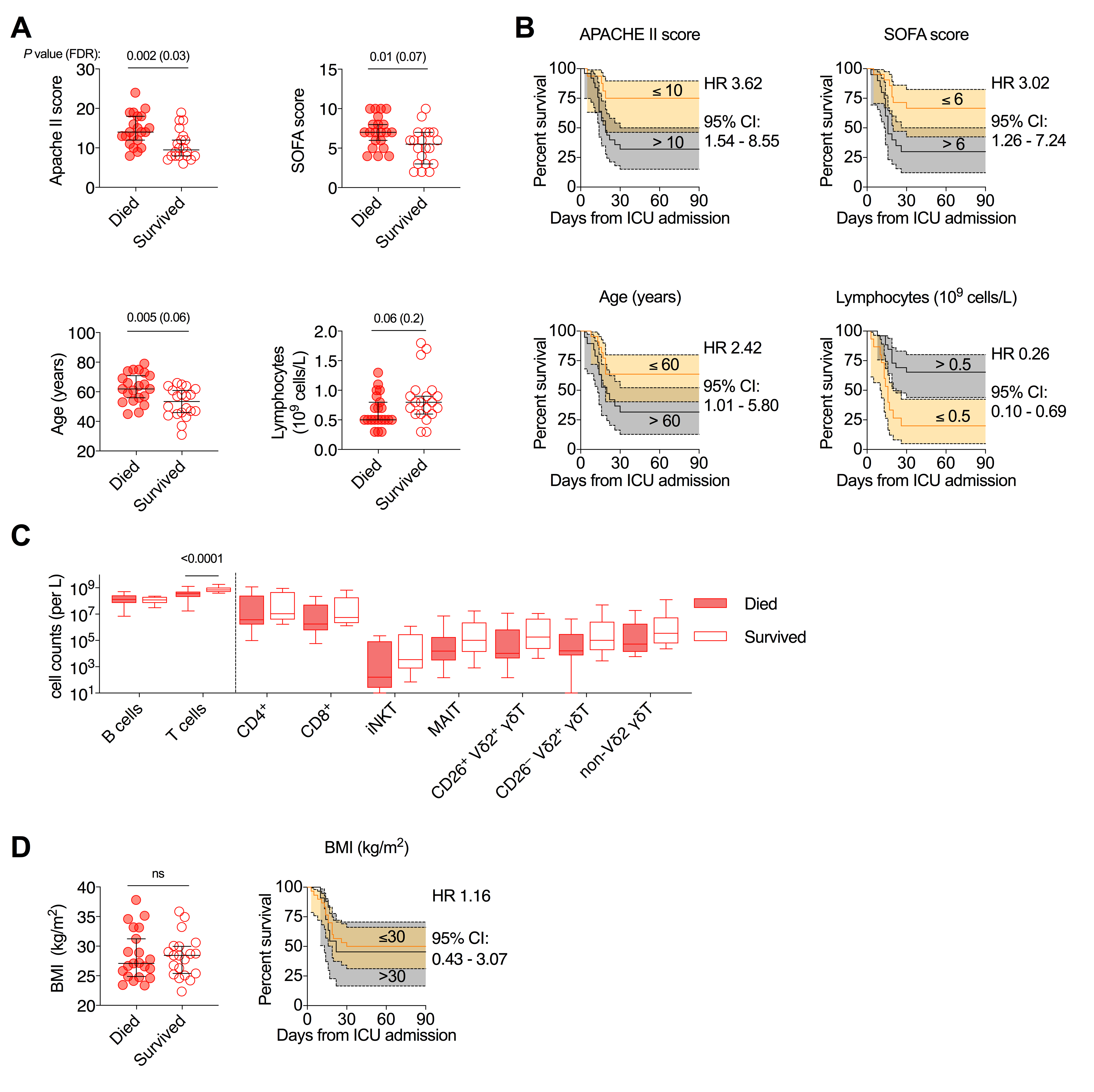

Supplement: S4 Fig — (A) Comparison of clinical measures between critically ill COVID-19 patients who died or survived. (B) Kaplan-Meier survival curves for clinical parameters from panel (A). (C) Absolute counts (per L) of the indicated lymphocyte population in critically ill COVID-19 patients who died or survived. (D) Comparison of body-mass index (BMI) between critically ill COVID-19 patients who died or survived (left), and Kaplan-Meier survival curve (right). Dots represent individual patients. Median ± 95% CI are shown. (A and D) Mann-Whitney U-test with Benjamini-Hochberg FDR calculation; (C) Two-way ANOVA with Sidak’s multiple comparison test. (TIFF) [file ppat.1009804.s004.tiff]

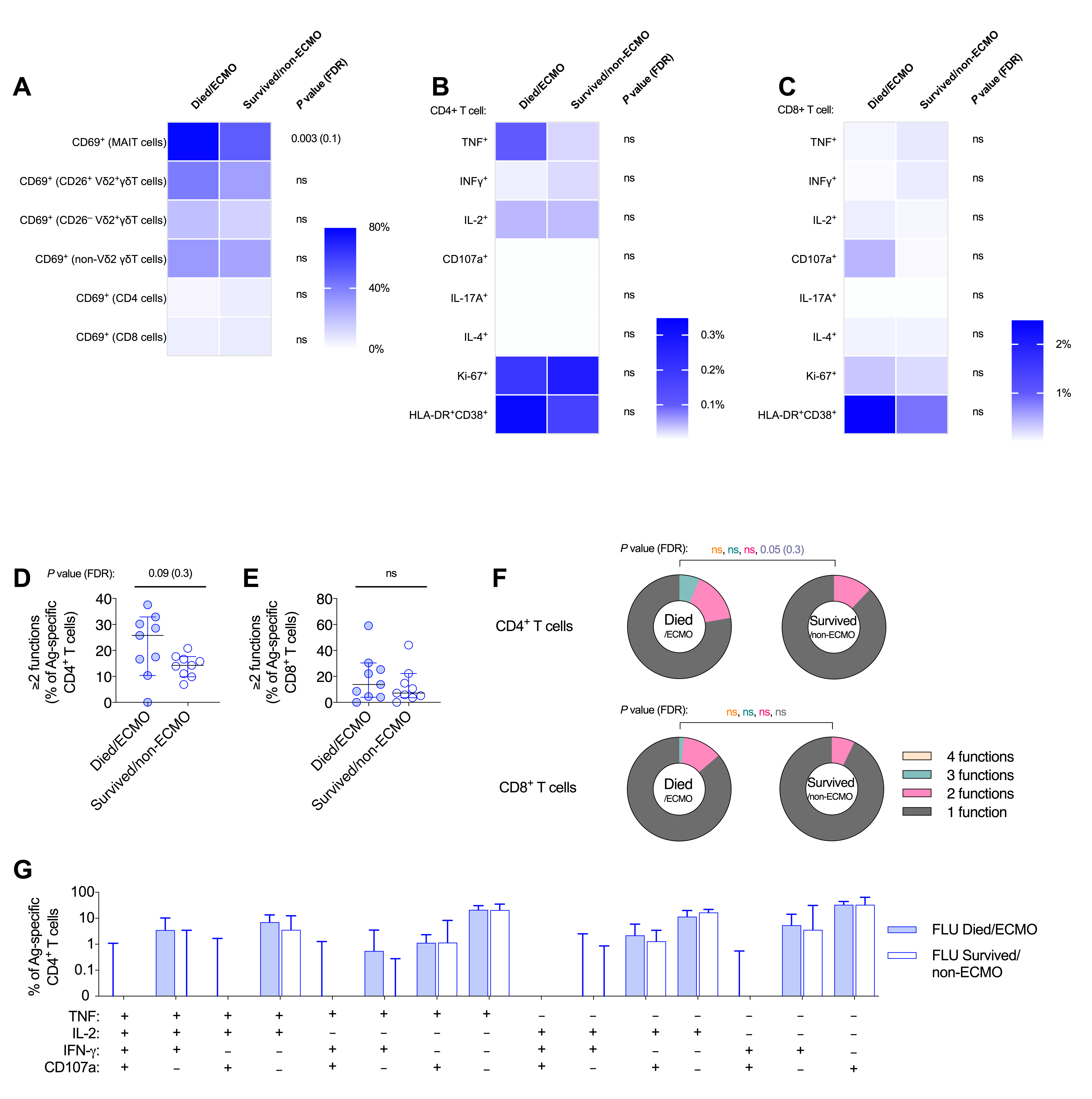

Supplement: S5 Fig — (A-C) Median expression of CD69 on the indicated T cell population (A), or the indicated cytokine or activation marker in CD4+ T cells (B) or CD8+ T cells (C) of critically ill influenza patients who died or required ECMO treatment versus those who did not. (D-E) Fraction of NP+M1-specific CD4+ T cells (D) CD8+ T cells (E) that are polyfunctional (≥2 cytokines produced) in critically ill influenza patients who died or required ECMO treatment versus those who did not. (F) Proportion of NP+M1-specific CD4+ T cells that produce 2, 3, or 4 cytokines (of CD107a, IFNγ, TNF, or IL-2) in critically ill influenza patients who died or required ECMO treatment versus those who did not. (G) Proportion of spike-specific CD4+ T cells that produce each of the indicated combinations of cytokines in critically ill influenza patients who died or required ECMO treatment versus those who did not. Dots represent individual patients. Median ± 95% CI are shown. (A to F) Mann-Whitney U-test with Benjamini-Hochberg FDR calculation. (TIFF) [file ppat.1009804.s005.tiff]

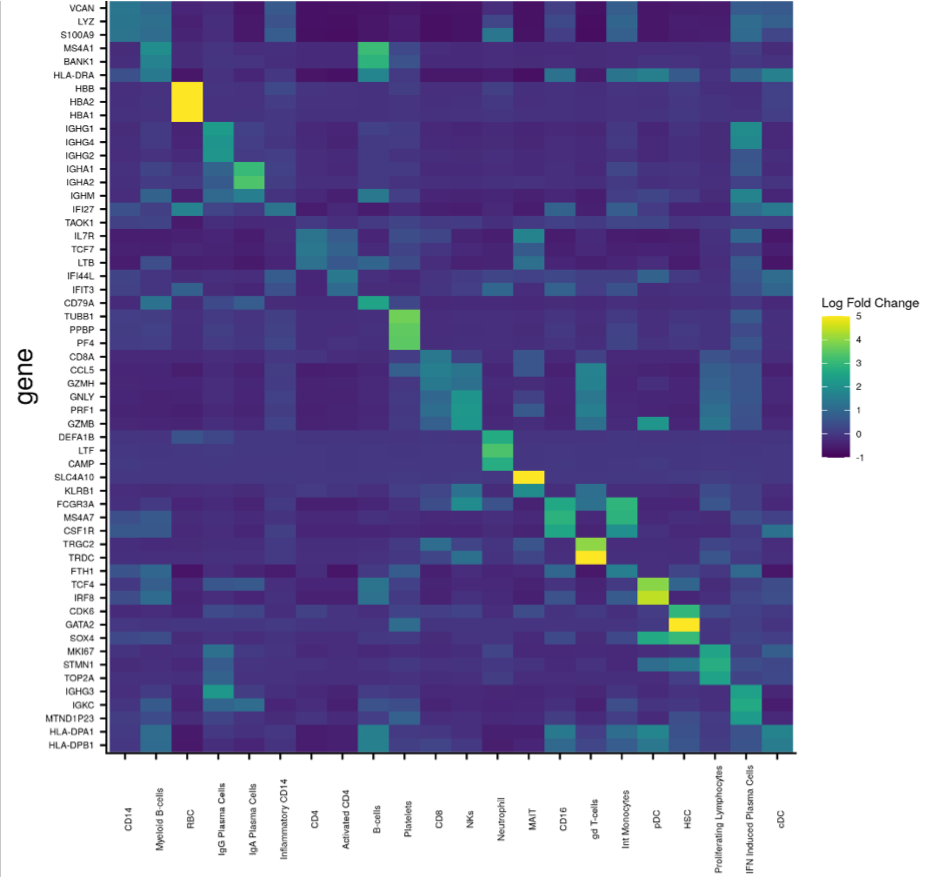

Supplement: S6 Fig — Top 3 genes for each cluster (used to annotate cell populations in Fig 6A). (TIFF) [file ppat.1009804.s006.tiff]

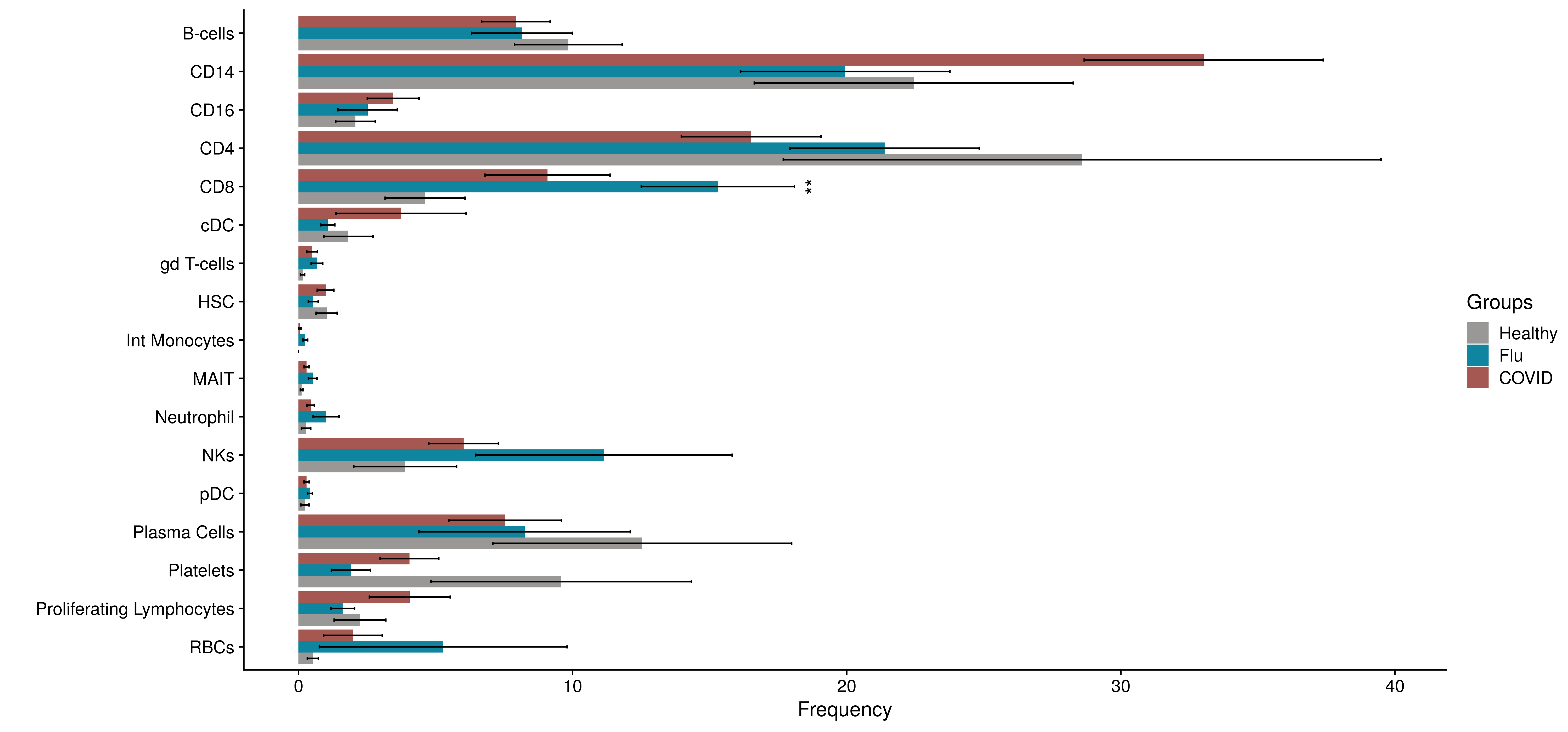

Supplement: S7 Fig — Relative cell proportions within each individual separated by disease condition. Statistical tests were conducted using Dirichlet regression between each condition. Mean ± standard deviation are shown. (TIFF) [file ppat.1009804.s007.tiff]

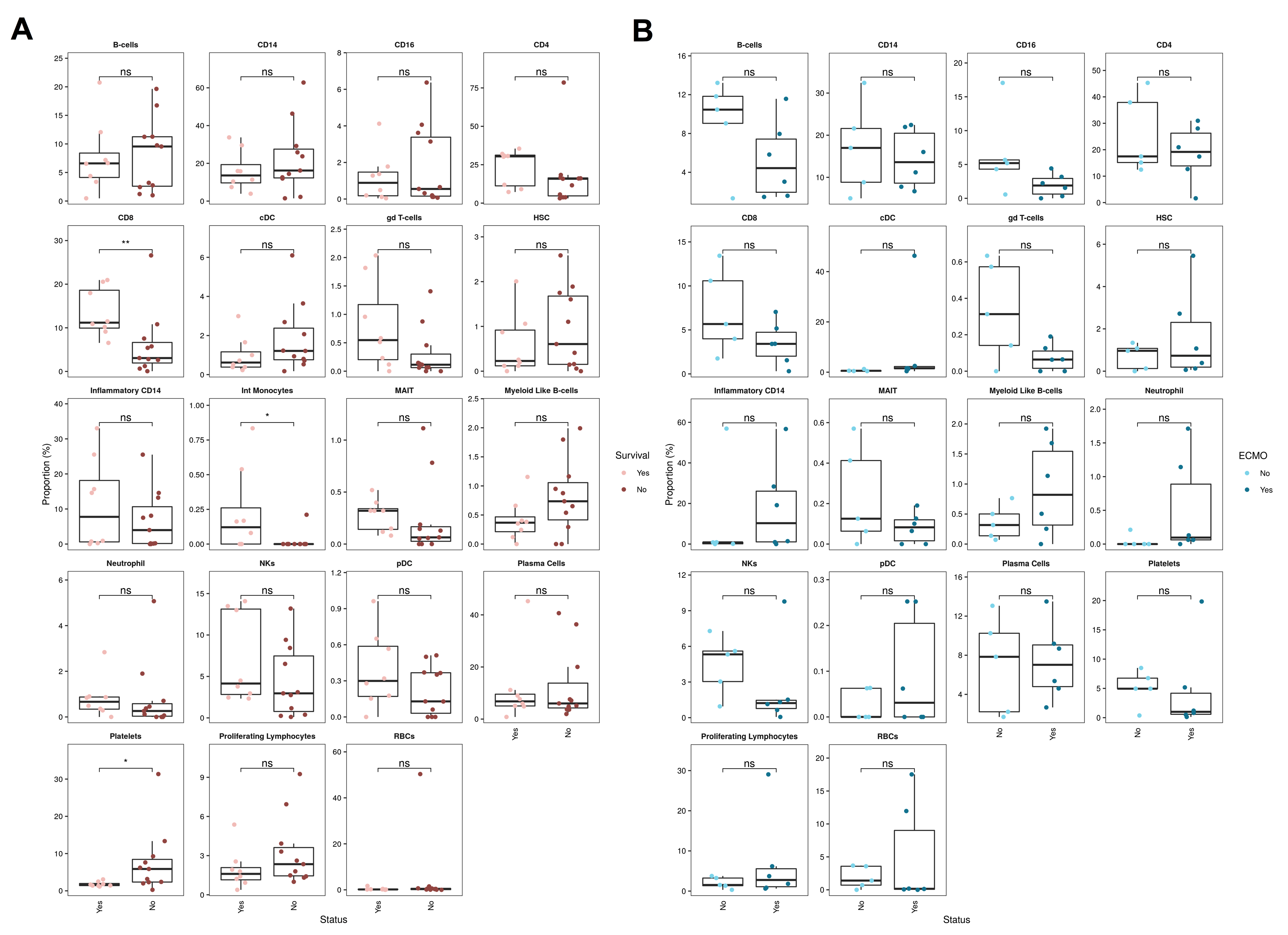

Supplement: S8 Fig — (A) Relative cell proportions within each COVID individual separated by survival and death. (B) Relative cell proportions within each influenza individual separated by ECMO and non-ECMO. Statistical tests were conducted using the Wilcoxon rank-sum test between each condition. Summary values were subsequently displayed using the boxplot. The box is equivalent to the interquartile range (IQR) with the median as the center, and whiskers correspond to the 25th percentile—1.5x IQR or the lowest value, and 75th percentile +1.5x IQR or the highest value. (TIFF) [file ppat.1009804.s008.tiff]

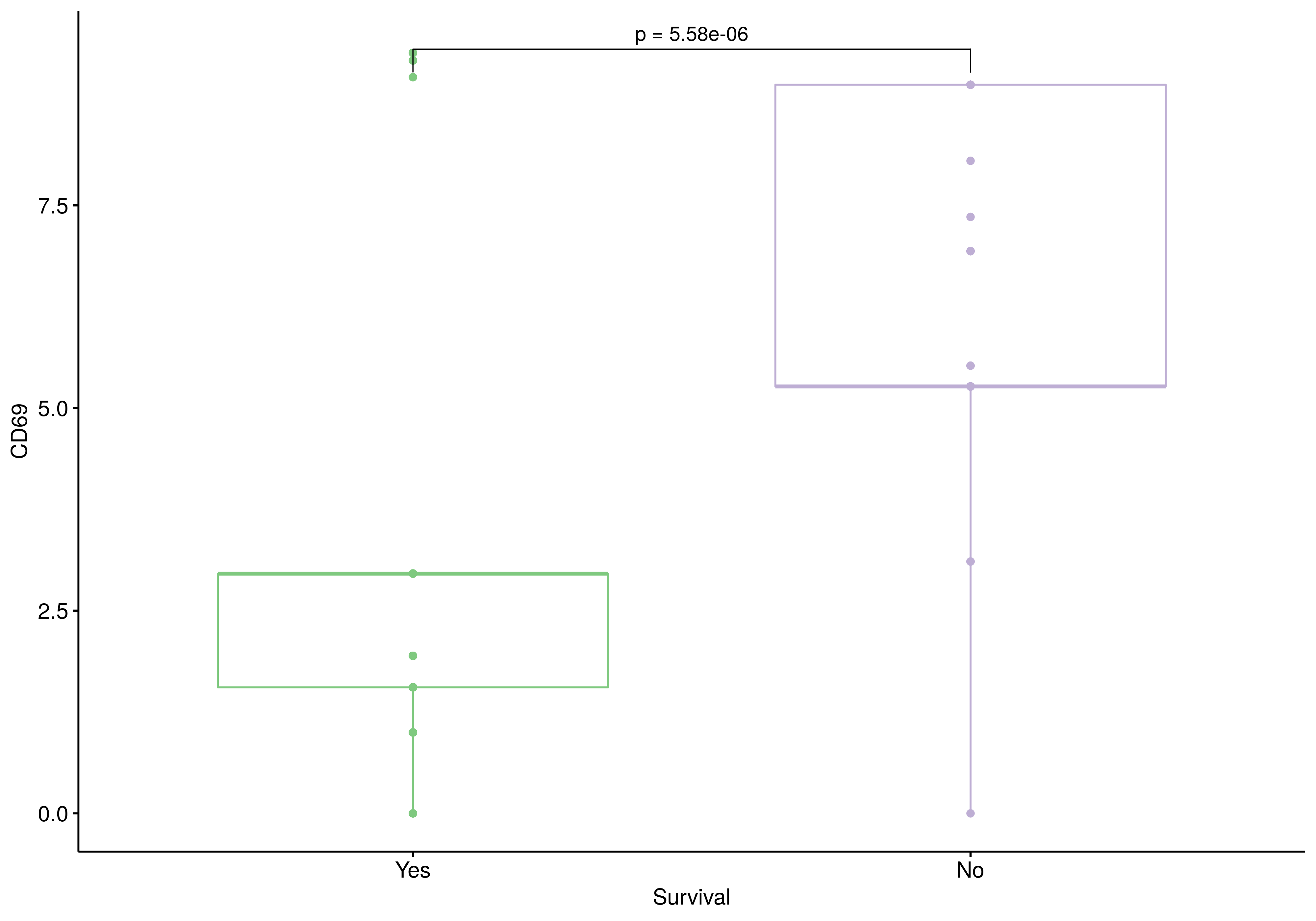

Supplement: S9 Fig — Expression of CD69 in survived and died COVID-19 patients. Significance was determined using the Wilcoxon test. (TIFF) [file ppat.1009804.s009.tiff]

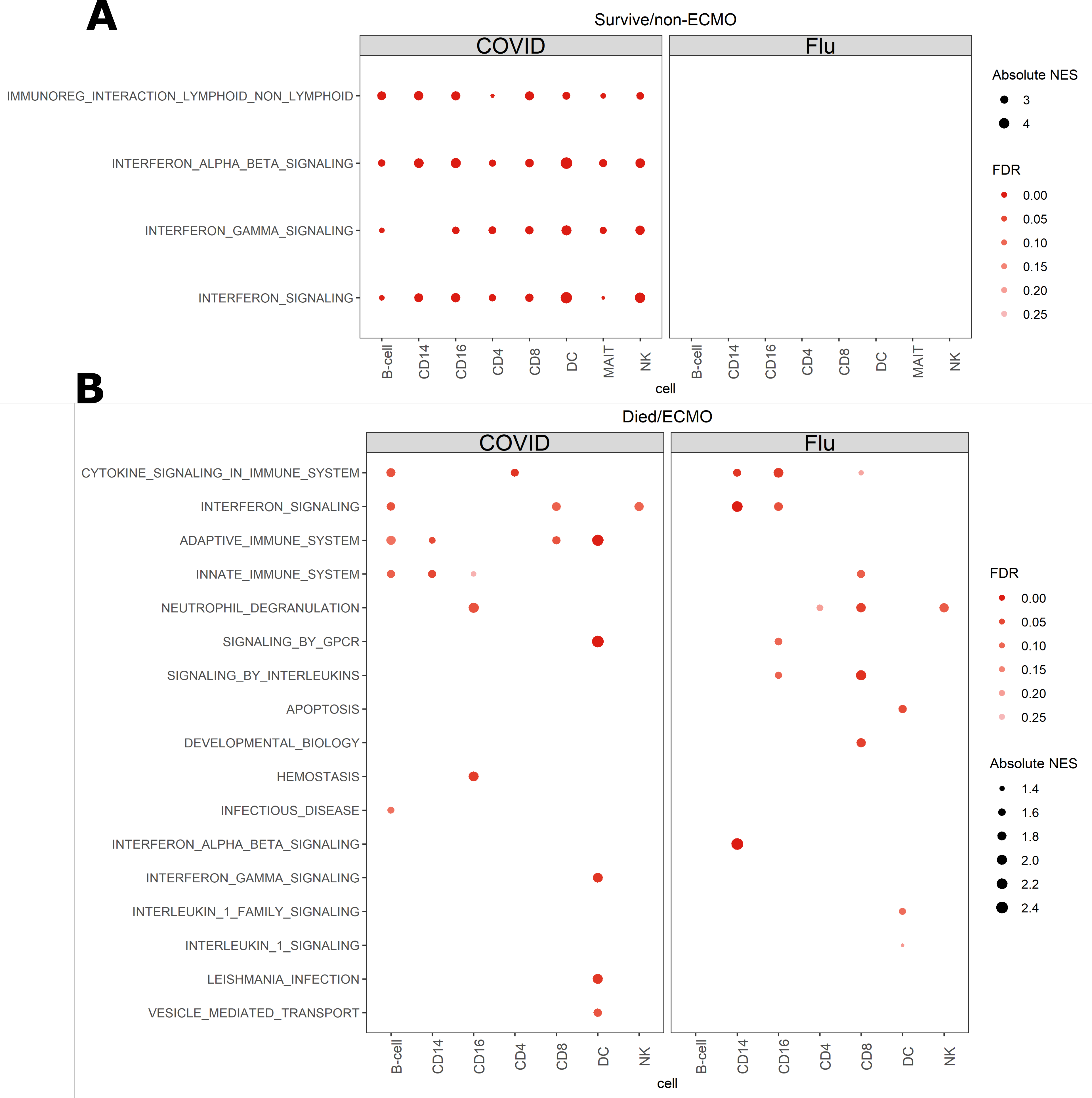

Supplement: S11 Fig — Curated dotplot of enriched pathways between (A) survived critically ill COVID patients versus survived/non-ECMO critically ill influenza patients and (B) died critically ill COVID patients versus died/ECMO critically ill influenza patients. Enriched pathways were obtained via reactome pathways identified via GSEA. FDRs were calculated based on q-values obtained from the hypergeometric test applied to the geneset followed by multiple hypothesis correction using the Benjamini-Hochberg method. NES: normalized enrichment score, FDR: false discovery rate. (TIFF) [file ppat.1009804.s011.tiff]
